# Deciphering shared attributes of plant long non-coding RNAs through a comparative computational approach

**DOI:** 10.1038/s41598-023-42420-7

**Published:** 2023-09-12

**Authors:** Vikash Kumar Yadav, Siddhi Kashinath Jalmi, Shalini Tiwari, Savita Kerkar

**Affiliations:** 1 School of Biological Sciences and Biotechnology, Goa University, Taleigao Plateau, Goa 403206 India; 2 Department of Biochemistry and Molecular Biology, Oklahoma State University, Stillwater, 74078 OK USA; 3 Present Address: National Institute of Plant Genome Research, Aruna Asaf Ali Marg, New Delhi, 110067 India

**Keywords:** Plant sciences, Bioinformatics, Genomic analysis, Sequencing

## Abstract

Over the past decade, long non-coding RNA (lncRNA), which lacks protein-coding potential, has emerged as an essential regulator of the genome. The present study examined 13,599 lncRNAs in *Arabidopsis thaliana*, 11,565 in *Oryza sativa*, and 32,397 in *Zea mays* for their characteristic features and explored the associated genomic and epigenomic features. We found lncRNAs were distributed throughout the chromosomes and the Helitron family of transposable elements (TEs) enriched, while the terminal inverted repeat depleted in lncRNA transcribing regions. Our analyses determined that lncRNA transcribing regions show rare or weak signals for most epigenetic marks except for H3K9me2 and cytosine methylation in all three plant species. LncRNAs showed preferential localization in the nucleus and cytoplasm; however, the distribution ratio in the cytoplasm and nucleus varies among the studied plant species. We identified several conserved endogenous target mimic sites in the lncRNAs among the studied plants. We found 233, 301, and 273 unique miRNAs, potentially targeting the lncRNAs of *A. thaliana*, *O. sativa*, and *Z. mays*, respectively. Our study has revealed that miRNAs, which interact with lncRNAs, target genes that are involved in a diverse array of biological and molecular processes. The miRNA-targeted lncRNAs displayed a strong affinity for several transcription factors, including ERF and BBR-BPC, mutually present in all three plants, advocating their conserved functions. Overall, the present study showed that plant lncRNAs exhibit conserved genomic and epigenomic characteristics and potentially govern the growth and development of plants.

## Introduction

Non-coding RNAs (ncRNAs) have been known to exist in higher eukaryotes for a long time, with most attention focused on small RNAs such as micro-RNA (miRNA) and small interfering RNA (siRNA). With increasing interest and focus on small RNAs, the list of these special classes of small regulatory RNAs keeps growing, e.g., Piwi-interacting RNA, repeat-associated siRNA, trans-acting siRNA, natural antisense transcript siRNA, heterochromatic siRNA, small scan RNA, and reveals their distinct functions in the regulation of biological processes in different organisms^1,2^. It has been firmly established that these small RNA molecules play pivotal roles in various regulatory processes such as transcription, post-transcription, and translation^1–3^.

Advanced sequencing technology and sensitivity have expedited the detection of novel transcripts, predominantly derived from the non-protein-coding region of the genome^2,4^. This has initiated to unearth a new class of ncRNA, long non-coding RNA (lncRNA). These transcripts are > 200 nucleotides in length and are known to modulate the biological activities throughout the realms of plants and animals^5–7^. The lncRNA has gained worldwide attention from researchers, which drives lncRNA identification across the kingdom^5^. The systematic examination of lncRNAs in plants, animals, and mammals has demonstrated that they play an essential role at the molecular level and contribute to processes such as transcription regulation, miRNA sponge, precursors of miRNAs and phasiRNAs, regulation of alternative splicing, and molecular cargos for protein transportation^6–9^. Despite influencing a wide range of biological processes at the molecular level, little is known about the mechanistic details of lncRNA function. However, several well-studied lncRNAs in plants and mammals have provided imperative clues about their functioning and mode of action^10–13^.

In plants, the multifaceted function of lncRNA showed their involvement in growth and development^14,15^, response to external stimuli^16^, role in stress response^17,18^, hormone signalling^17,19^, nutrient uptake and homeostasis^20^, transcriptional regulation^18,21^, epigenetic regulation^15^ etc. Following are some well-known examples of lncRNAs biological function in plants. In rice, a lncRNA named long-day-specific male-fertility-associated RNA (LDMAR) is required for normal pollen development of plants grown under long-day conditions. A single nucleotide mutation causes changes in LDMAR's structure, leading to reduced transcription, causing premature cell death in anthers and photoperiod-sensitive male sterility^14^. A study in Sea buckthorns revealed two lncRNA, LNC1 and LNC2, acting as miRNA target mimics to influence anthocyanin content via SPL9 and MYB114 regulation and revealed their role in anthocyanin content and fruit ripening^22^. A group of well-characterized antisense lncRNAs transcribing from the floral-repressor locus (FLC), called COOLAIR adopts various conformational structures governing the *FLC* transcriptional output in response to warm and cold conditions^16^. In cotton, lncRNA973 has been shown to enhance salt tolerance by regulating the expression of several salt stress-related genes^18^. In wheat, lncR9A, lncR117 and lncR616 were shown to control the level of CDS1 by modulating the expression of tae-miR398 and improving the cold resistance mechanism in winter wheat^21^. Another study in rice revealed the role of lncRNA, TCONS_00021861 YUCCA7 gene by modulating the level of miR528-3p, which leads to an increased level of IAA and confers drought tolerance^17^. In *Z. mays*, researchers identify GIBBERELLIN-RESPONSIVE lncRNA (GARR2) derived from a Gypsy LTR retrotransposon^19^. GARR2 editing showed GA-induced effects, altering GA-related genes and affect on primary auxin response. GARR2 interacted with ZmUPL1, a HECT ubiquitin-protein ligase. GARR2 influenced ZmUPL1 levels in GA response, revealing lncRNA roles in GA-modulated plant height^19^. Franco-Zorrilla and colleagues showed that in *A. thaliana*, lncRNA INDUCED BY PHOSPHATE STARVATION1 (IPS1) governs the Pi homeostasis by modulating the expression of PHO2 through sequestering the miR-399^20^. In *A. thaliana*, winter cold triggers epigenetic repression of FLOWERING LOCUS C (FLC), via cold-induced histone modification involving a lncRNA, COLD ASSISTED INTRONIC NONCODING RNA (COLDAIR), which interacts and recruits PRC to FLC^15^.

The growing list of lncRNAs across different plant species vouches for their functions in plant growth, development, and stress response, necessitating the understanding of features associated with lncRNAs^23,24^. This knowledge gap has spurred us to systematically analyze plant lncRNAs to determine their conserved features, which might help us understand their biological significance. In the present study, we aimed to determine the general characteristics of lncRNAs in *A. thaliana*, *O. sativa*, and *Z. mays* and explore their genomic and epigenomic-associated features. We examined the subcellular localization of lncRNAs and studied the interaction network of transcription factors (TFs) and lncRNA. Furthermore, we systematically analyzed the association of transposable elements (TEs) with lncRNAs in plants. Finally, we investigated the lncRNA-miRNA-mRNA interactome network to explore the role of lncRNA in biological and cellular processes. Our study will provide novel insights into the characteristics and conserved features associated with plant lncRNAs.

## Results

### Genomic distributions and general characteristics of plants lncRNA

**Figure 1:**
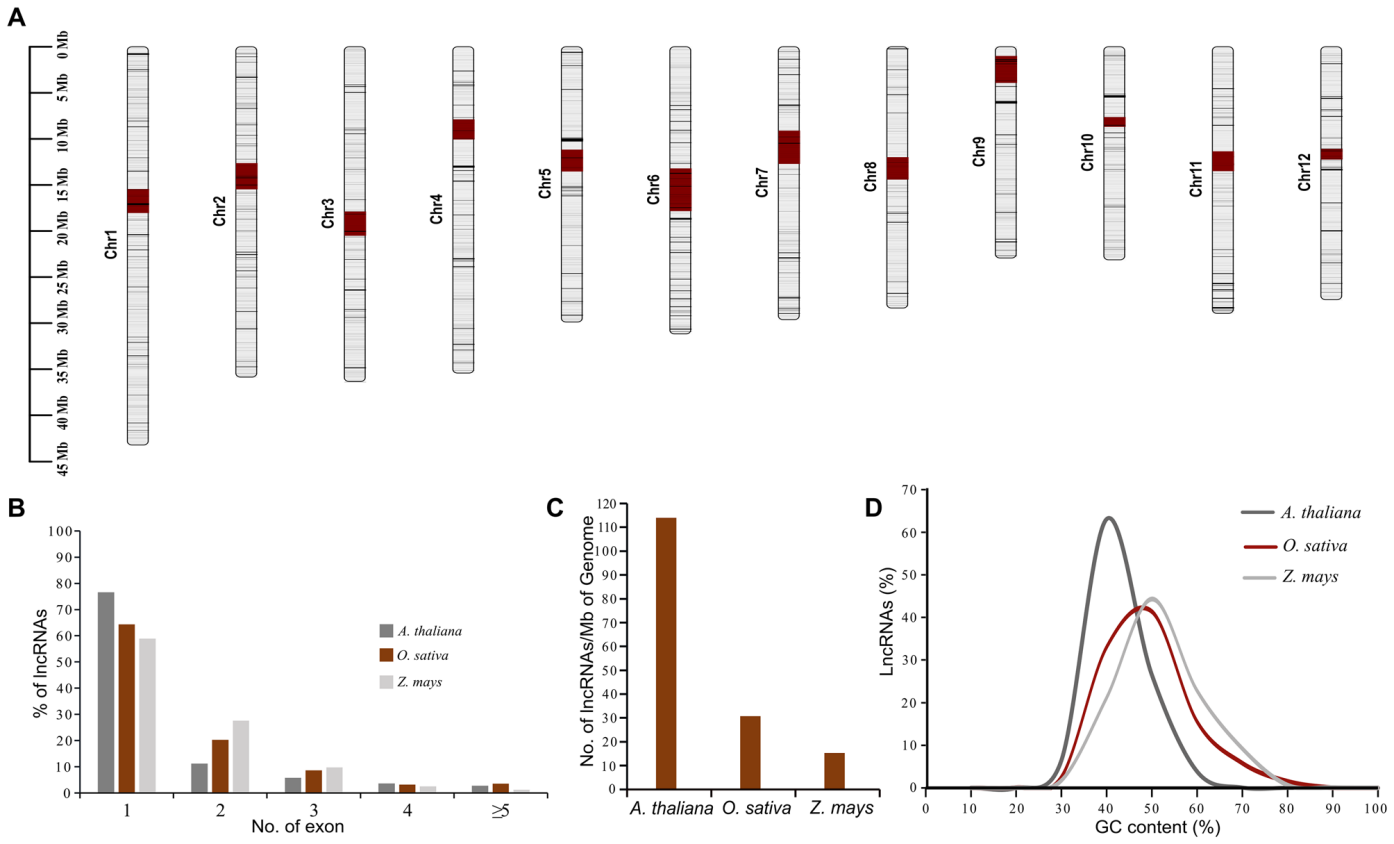
General characteristics of plant lncRNAs. (**A**) Distribution of lncRNAs along the chromosomes of *O. sativa.* Genomic distribution of lncRNAs for *A. thaliana* and *Z. mays* are shown in Fig. S1. (**B**) Bar graph showing the number of exons per lncRNA transcript in *A. thaliana*, *O. sativa*, and *Z. mays*. The majority of lncRNA transcripts are mono-exonic in all three studied plants. (**C**) The bar graph representing the density of lncRNAs per Mb of the genome shows that the smaller genome has more lncRNA density than the larger genome. (**D**) GC content of lncRNAs in *A. thaliana*, *O. sativa*, and *Z. mays*.

The lncRNAs of *A. thaliana*, *O. sativa*, and *Z. mays* were retrieved from the public repository PLncDB V2.0, containing an extensive catalogue of plant lncRNAs^23^. A total of 13,599, 11,565, and 32,397 lncRNAs were obtained for *A. thaliana*, *O. sativa*, and *Z. mays*, respectively. We analyzed lncRNA distribution along the chromosome to determine whether lncRNAs were transcribed from any preferential region. The distribution pattern revealed that lncRNAs are distributed throughout the chromosomes, chromosomal arms, telomeric and centromeric regions in studied plants (Fig. 1A; Fig. S1). Further, the lncRNAs show no preferential distribution pattern based on chromosome size among the studied plant. The median length of lncRNA transcripts were 330, 579, and 636 nucleotides (Fig. S2A), while the average length of lncRNAs was 765, 2539, and 2438 nucleotides in *A. thaliana*, *O. sativa*, and *Z. mays*, respectively. The size distribution of lncRNA transcripts showed that *A. thaliana* has a higher percentage (87.3%) of small transcripts (< 1 Kb), followed by *Z. mays* (66.3%) and *O. sativa* (59%) and both *O. sativa* and *Z. mays* contain lncRNA transcripts > 10 Kb approximately twice that of *A. thaliana*.

A total of 23.4%, 35.6%, and 41.0% of lncRNAs were spliced in *A. thaliana*, *O. sativa*, and *Z. mays*, respectively, which means that *A. thaliana* had the most mono-exonic lncRNAs and *Z. mays* had the most multi-exonic lncRNA transcripts (Fig. 1B). The lncRNAs showed an average of 1.4, 1.7, and 1.6 exons per gene, and the median length of the exon was 246, 239, and 268 nucleotides in *A. thaliana*, *O. sativa*, and *Z. mays*, respectively (Fig. S2B). However, the percentage of smaller exons (< 200 bp) is higher in *O. sativa* and *Z. mays* than in *A. thaliana*. The increase in the average length of the lncRNA gene from *A. thaliana* to *O. sativa* and *Z. mays* is complemented by the high number of exons, hence the intron (Fig. 1B).

Furthermore, we investigated the density of lncRNAs in the genome and found that approximately 114, 30.8, and 15.3 lncRNA transcripts were present per Mb of the genome in *A. thaliana*, *O. sativa*, and *Z. mays*, respectively. LncRNA density was negatively correlated with genome size but positively correlated with the number of PCGs per Mb of the genome (Fig. 1C and Fig. S3). The GC content of lncRNAs showed that *Z. mays* lncRNAs have higher GC content, *A. thaliana* showed lower GC content, while the *O. sativa* lncRNAs showed GC content in between that of *A. thaliana* and *Z. mays* (Fig. 1D). Overall, our analyses revealed that the complexity of lncRNA transcripts (length, exon, and intron numbers) increases with the complexity of genomes (genome size).

### Localization of lncRNAs revealed predominant localization in the nucleus and cytoplasm

**Figure 2:**
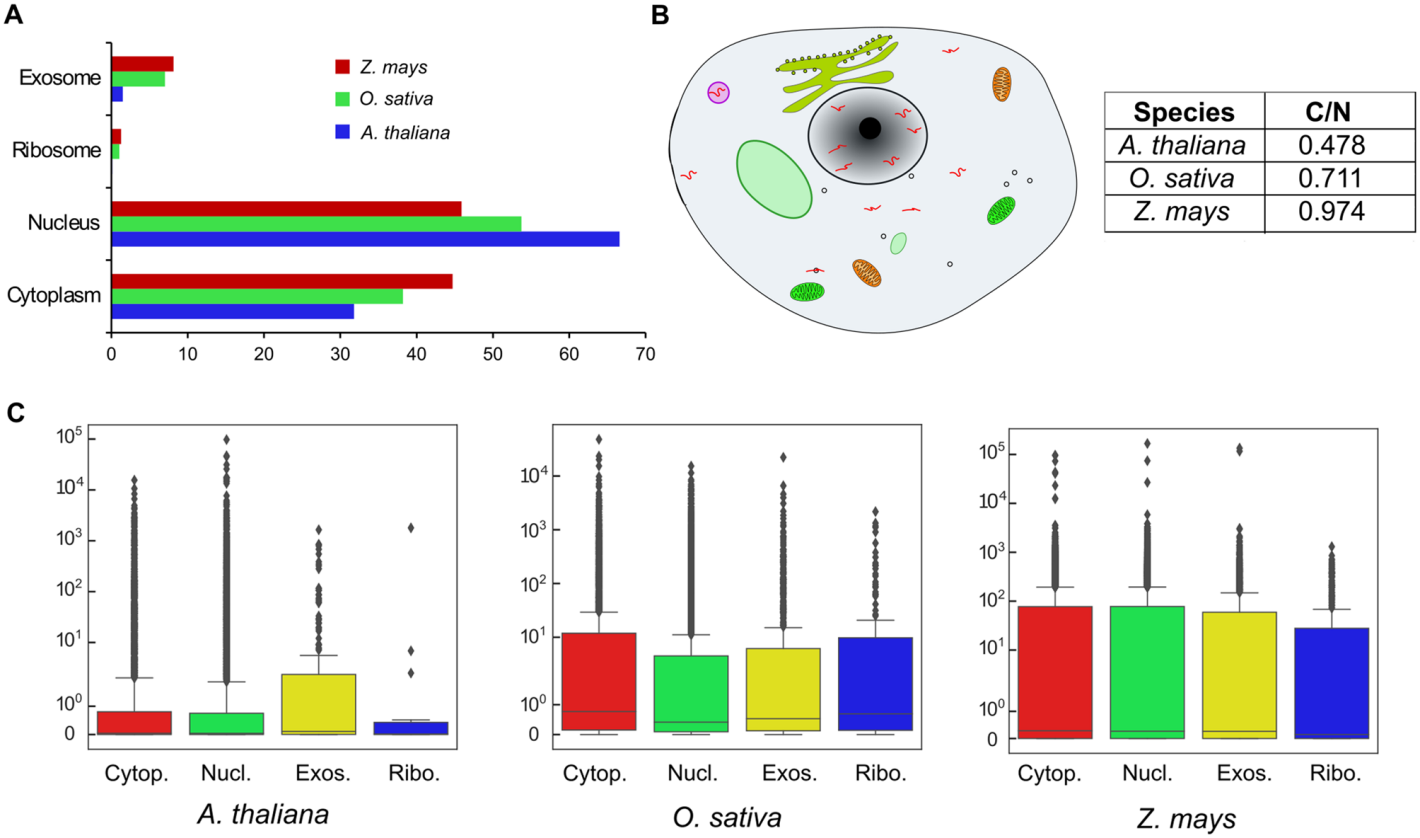
Subcellular localization of lncRNAs and their expression profile (**A**) Determination of lncRNA localization in different subcellular fractions showing the majority of lncRNAs localized in the nucleus and cytoplasm. (**B**) The distribution ratio of total lncRNAs in the cytoplasm and nucleus (C/N ratio). (**C**) Box plot showing the average expression profile of lncRNAs localized in the cytoplasm, nucleus, ribosome, and exosome.

As lncRNA act as functional molecules in almost every cellular activity, it is essential to study their subcellular localizations, which possess vital information associated with their biological roles^7,24^. We determined the subcellular localizations of lncRNAs and classified them into four categories: cytoplasm, nucleus, ribosome, and exosome. In *A. thaliana*, 31.8% and 66.6% of lncRNAs were localized in the cytoplasm and nucleus, respectively, while in *O. sativa*, 38.2% and 53.7% and in *Z. mays*, 44.7% and 45.9% of lncRNAs were localized in the cytoplasm and nucleus, respectively (Fig. 2A). In *Z. mays* and *O. sativa*, 8.1% and 7.0% of lncRNAs localized in the exosome, respectively, as compared to that of 1.5% in *A. thaliana* (Fig. 2A). LncRNA localization in the ribosome were predicted to be least, revealing their predominant localization in the cytoplasm and nucleus, which reflects their apparent site of action.

The lncRNAs are transcribed in the nucleus but potentially exported and localized in different subcellular compartments to perform specific functions (Fig. 2B). The distinct subcellular localization of lncRNAs enables them to perform diverse functions by facilitating interactions with other functional molecules. The cytoplasmic/nuclear (C/N) ratio of total lncRNAs was found to be 0.48, 0.71, and 0.97 in *A. thaliana*, *O. sativa*, and *Z. mays*, respectively (Fig. 2B). In *A. thaliana*, the localization of lncRNAs in the nucleus is almost twice than in the cytoplasm, while in *Z. mays*, they are equally localized in the nucleus and cytoplasm, suggesting that either the distribution of lncRNA in the cytoplasm and nucleus could be very dynamic, or their abundance and subcellular localization may vary among plant species (Fig. 2B).

To investigate whether the lncRNAs showing distinct subcellular localization have any correlation with their expression pattern, we plotted the average expression values of lncRNAs (extracted from PLncDB V2.0). Comparing the expression profiles of lncRNAs in the four subcellular compartments, we found that there is no typical pattern in the expression profile of lncRNAs localized in different subcellular fractions in *A. thaliana*, *O. sativa*, and *Z. may* (Fig. 2C). However, the expression levels of lncRNAs were found to be higher in *O. sativa* and *Z. mays* as compared to *A. thaliana* (Fig. 2C). This suggested that their subcellular localization does not influence the expression level of lncRNA, and their expression level remains the same in different subcellular organelles (Fig. 2C).

### Genomic and epigenetic features associated with plant lncRNAs

**Figure 3:**
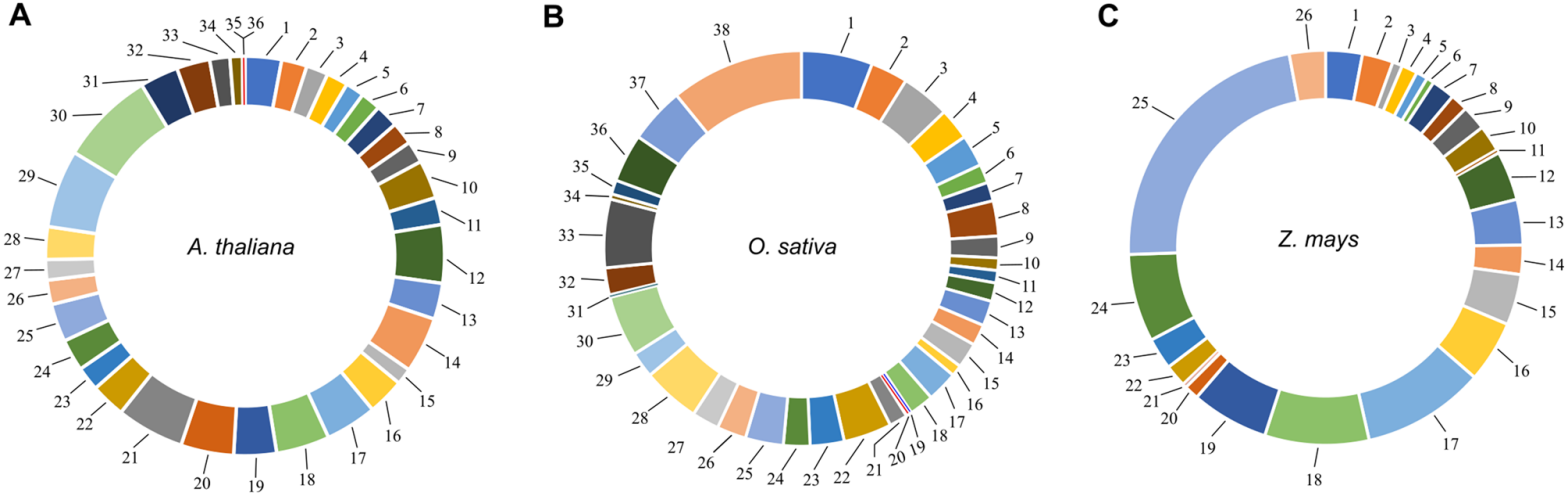
Epigenomic characteristics of lncRNAs. Mapping lncRNAs onto different epigenetic states reveals the enrichment of specific epigenetic marks and genomic features associated with lncRNA transcribing regions. (**A**) In *A. thaliana*, lncRNAs are predominantly enriched in epigenetic states 21, 29, and 30. (**B**) *O. sativa* lncRNAs show enrichment in epigenetic states 1, 33, and 38. (**C**) *Z. mays* lncRNAs showing enrichment in epigenetic states 16, 17, 18, 19, 24, and 25.

Epigenetic signatures associated with the genome significantly impact the transcriptional ability and accessibility of genomic loci. To understand whether any preferential and conserved epigenetic marks are associated with the lncRNA, we conducted a correlation study between lncRNA and their genome, divided into distinct regions based on epigenomic features. The PCSD database of epigenomic signatures divides the genomes of *A. thaliana*, *O. sativa*, and *Z. mays* into 36, 28, and 38 different epigenetic states, respectively, based on various epigenetic and genomic features^25^. Using the PCSD database web tool, we investigated the epigenetic and genomic features associated with lncRNA in three studied plant species. In *A. thaliana*, epigenetic states 21, 29, and 30 predominantly overlapped with lncRNA transcribing regions, which are mainly enriched for genomic features such as the promoter and intergenic regions of the genome (Fig. 3A). Furthermore, these epigenetic states showed weak and rare signals for epigenetic marks. These epigenetic states are also enriched for ncRNAs like miRNA, snRNA, and snoRNA. Epigenetic state 21 represents the chromatin-accessible regions, as determined by DNase I hypersensitivity and ATAC-seq, and provides the binding site for several TFs (PIF3/4, PHYB, PhyA, FHY1, FRS12, CCA1, SOC1, LFY, AP1/2/3, KAN1, PPD2, SPCH, ARR10, WRKY18/33/40, SPL7).

In *O. sativa*, lncRNA transcribing regions were mainly represented by epigenetic states 1, 33, and 38, which overlap with promoters, coding regions, intergenic regions, and TE regions (Fig. 3B). Epigenetic states 1 and 38 showed weak and rare signals for epigenetic marks whereas, epigenetic state 33 is enriched for DNA methylation, H3K9me2, and showed accessibility for MNase. In *Z. mays*, the majority of the lncRNA transcribing regions were represented by epigenetic states 16, 17, 18, 19, 24, and 25, which primarily overlapped with the intergenic region, repeat, and centromeric part of the genome (Fig. 3C). These regions were enriched for DNA methylation, H3K9me2, and MNase. Interestingly, the most prominent epigenetic state 25, which overlaps with lncRNA transcribing regions, showed rare signals for epigenetic marks similar to *A. thaliana* and *O. sativa*. All these enriched ESs of *Z. mays* also showed enrichment for the TFs CCA1b, RAD51 and for the epigenetic marks H4K5ac and H3K56ac. This analysis highlights a few conserved features of lncRNA transcribing regions that overlap with genic and intergenic regions. The result also suggests that lncRNA could also act as trapping molecules for TFs to facilitate gene regulation.

### Relationship of lncRNA with transposable elements

**Figure 4:**
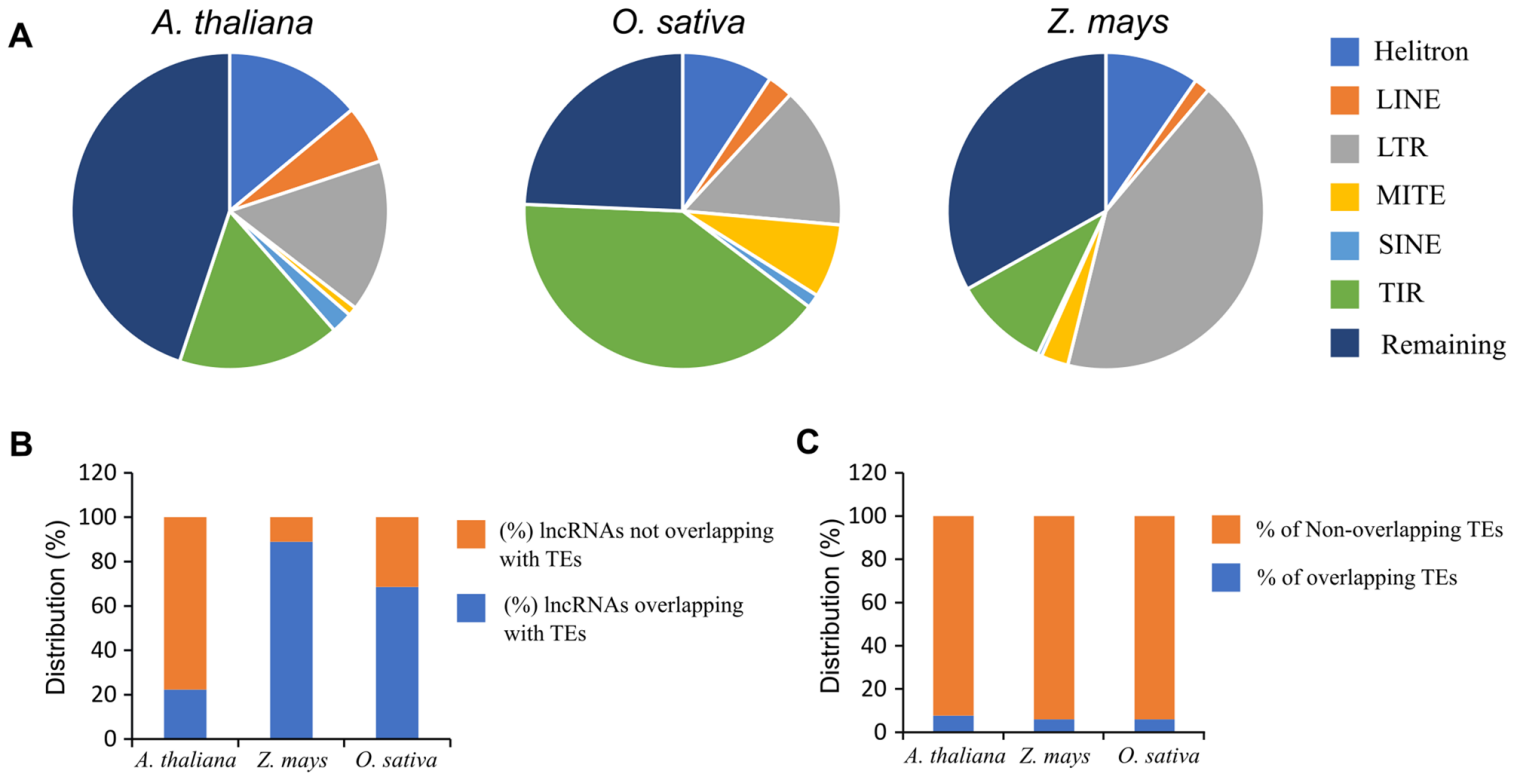
Association of lncRNAs with transposable elements (**A**) Association of lncRNAs with different categories of TEs (Helitron, LINE, LTR, MITE, SINE, TIR, and others). (**B**) The percentage of lncRNAs overlapping with TEs revealed lncRNAs in *A. thaliana* with the least overlap (22.2%), while those in *Z. mays* with the maximum overlap (88.9%) with TEs. (**C**) The percentage distribution of TEs in lncRNA transcribing regions shows similar percentages in the studied plant.

TEs are characteristic features of higher organisms, despite their diverse genomes, including size, ploidy, and heterozygosity. Hence, to understand the systematic association of lncRNAs with the parts of the genome that encode TEs, we first determined the distribution of different types of TEs in lncRNA transcribing regions. We used the APTE database of TEs, which provides systematically identified TEs in many plant species using uniform parameters and a standardized catalogue of TE annotation^26^. We found an enrichment of Helitron TE in lncRNA transcribing regions, compared to their distribution across the genome (Fig. 4A, Fig. S4). The enrichment of the Helitron family of TEs in all three plant species suggests that this might be a conserved feature of plant lncRNAs. Similarly, the terminal inverted repeats (TIR) class of TEs showed consistent depletion in lncRNA transcribing regions compared to its distribution across the genome (Fig. 4A, Fig. S4). In *Z. mays*, the long terminal repeats (LTR) family also showed enrichment in lncRNA transcribing regions but not in *A. thaliana* and *O. sativa* (Fig. S4). We calculated the percentage of lncRNAs that overlapped with TEs and discovered that in *A. thaliana*, 22.2% of lncRNAs overlapped with TEs, while in *O. sativa* and *Z. mays*, 68.5% and 88.9% of lncRNAs were overlapping with TEs in the genome (Fig. 4B). The number of TEs significantly higher in *O. sativa* (> 10x) and *Z. mays* (> 20x) compared to the *A. thaliana* positively link the TEs number with lncRNAs overlapping with TEs. Interestingly, the overall TE percentage distribution in lncRNA transcribing regions seems very uniform (~ 6–8%), even though the density of TEs varies among the studied plant (Fig. 4C).

### LncRNAs association with miRNA and their role in governing biological processes

**Figure 5:**
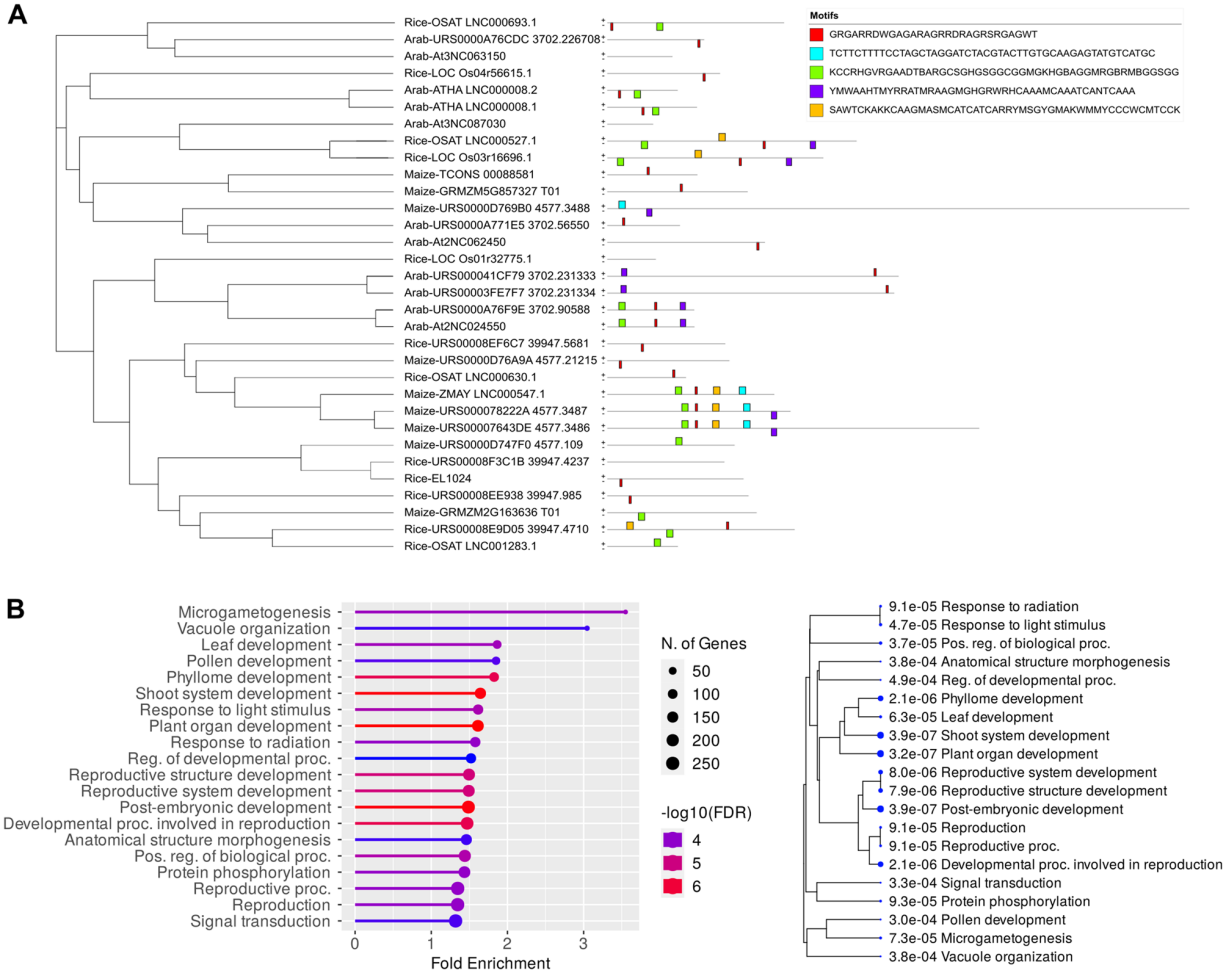
Conserved features and biological processes associated with lncRNAs. (**A**) The cladogram shows the relatedness among the lncRNAs of studied plants that possess the endogenous target mimic. The lncRNAs possessing the endogenous target mimic reveal conserved motifs, as determined by MEME. (**B**) GO enrichment analysis showing the top 20 biological processes derived from lncRNA-miRNA-mRNA interactome in *A. thaliana*. The significant GO enrichment plots at *p* > 0.05 are depicted using ShinyGO v0.76. The fold enrichment of GO terms represents how drastically particular pathway genes are overrepresented. Hierarchical clustering shows the clustering of similar biological functions. Bigger dots indicate more significant *p*-values.

LncRNAs act as miRNA decoys or sponges, which is mediated by interrupted complementarity between the miRNA and lncRNA^20^. We identified interrupted complementarity at the expected cleavage site using psMimic^27^. We found 11 lncRNAs possess endogenous target mimic sites for ten different miRNAs (Table S1A) in *A. thaliana*. In the case of *O. sativa*, 12 lncRNAs possess endogenous target mimic sites for 16 different miRNAs (Table S1B), while in *Z. mays*, 9 lncRNAs possessed endogenous target mimic sites for 16 different miRNAs (Table S1C). The presence of endogenous target mimic sites in all three studied plants suggests it is a common mechanism for fine-tuning miRNA activity in cellular environments for gene regulation. The cladogram between the lncRNAs possessing endogenous target mimics of the studied plant revealed relatedness among the lncRNAs (Fig. 5A). Further, motifs identification in the lncRNAs possessing the endogenous target mimic revealed conserved motifs in plants lncRNAs, as determined by MEME (Fig. 5A). The miRNA targeted by lncRNA endogenous mimics also showed relatedness among the studied plants (Fig. S5). The conserved miRNAs targeted by the endogenous target mimic site of lncRNAs in studied plants suggested that these lncRNAs might have conserved functions in the plant system (Fig. 5A, Fig. S5).

LncRNAs can modulate small RNAs or transcriptional regulatory proteins in the cellular system and regulate gene expression. We determined the miRNAs that potentially target and cleave lncRNAs using psRNATarget. A total of 233, 301, and 273 unique miRNAs were found to target the lncRNAs, representing 54.4%, 40.8%, and 84% of the total mature miRNAs reported in the database (https://mirbase.org) in *A. thaliana*, *O. sativa*, and *Z. mays*, respectively (Supplementary File 1). Identified miRNAs targeting lncRNAs for cleavage could potentially affect the impact of miRNAs on the target genes by modulating the availability of miRNAs for target genes. We identified the target genes for these miRNAs to understand the role of these lncRNA-miRNA-mRNA networks in biological and developmental processes. To determine the over-represented biological processes, we performed enrichment analysis on lncRNA-miRNA-targeted genes (Supplementary File 2).

The GO enrichment analysis in *A. thaliana* showed processes like micro gametogenesis, pollen development, organ development, reproductive organs, response to light, and signal transductions (Fig. 5B). The GO enrichment analysis in *O. sativa* showed the role of lncRNA in flower development, cellular response to stimuli, regulation of transcription, developmental processes, regulation of macromolecule biosynthetic processes, lignin catabolic and metabolic processes, etc. (Fig. S6A). Furthermore, GO enrichment analysis in *Z. mays* showed enrichment of biological processes such as DNA repair, reproductive processes, lignin metabolic and catabolic processes, and DNA biosynthesis (Fig. S6B). The lncRNA-miRNA-mRNA network potentially regulates a wide range of functions, some of which are conserved among the three studied species, such as reproduction-associated processes, while lignin catabolic and metabolic processes in *O. sativa* and *Z. mays* imply their biological significance (Fig. 5B, Fig. S6).

The GO-enrichment for molecular function revealed that the sequence-specific DNA binding, DNA-binding transcription factor activity and transcription regulator activity are conserved in all the three-studies plants (Fig. S7, Supplementary File 3). In *A. thaliana* and *O. sativa*, molecular functions like ADP binding, kinase activity, oxidoreductase activity, transcriptional cis-regulatory region binding, and transferase activity are enriched (Fig. S7, Supplementary File 3). Molecular functions such as ATP-dependent activity, hydrolase activity, oxidoreductase activity, and pyrophosphatase activity have been found to be enriched in *O. sativa* and *Z. mays* (Fig. S7, Supplementary File 3). The GO-enrichment for the cellular component does not show any enrichment for *A. thaliana* and *O. sativa*, however in *Z. mays*, its enrichment as a component of the replisome, transcription regulator complex, RNA polymerase complex etc. revealed their significance in genome regulation (Fig. S8).

### LncRNAs revealed conserved binding sites for TFs, ERF, and BBR-BPC

**Figure 6:**
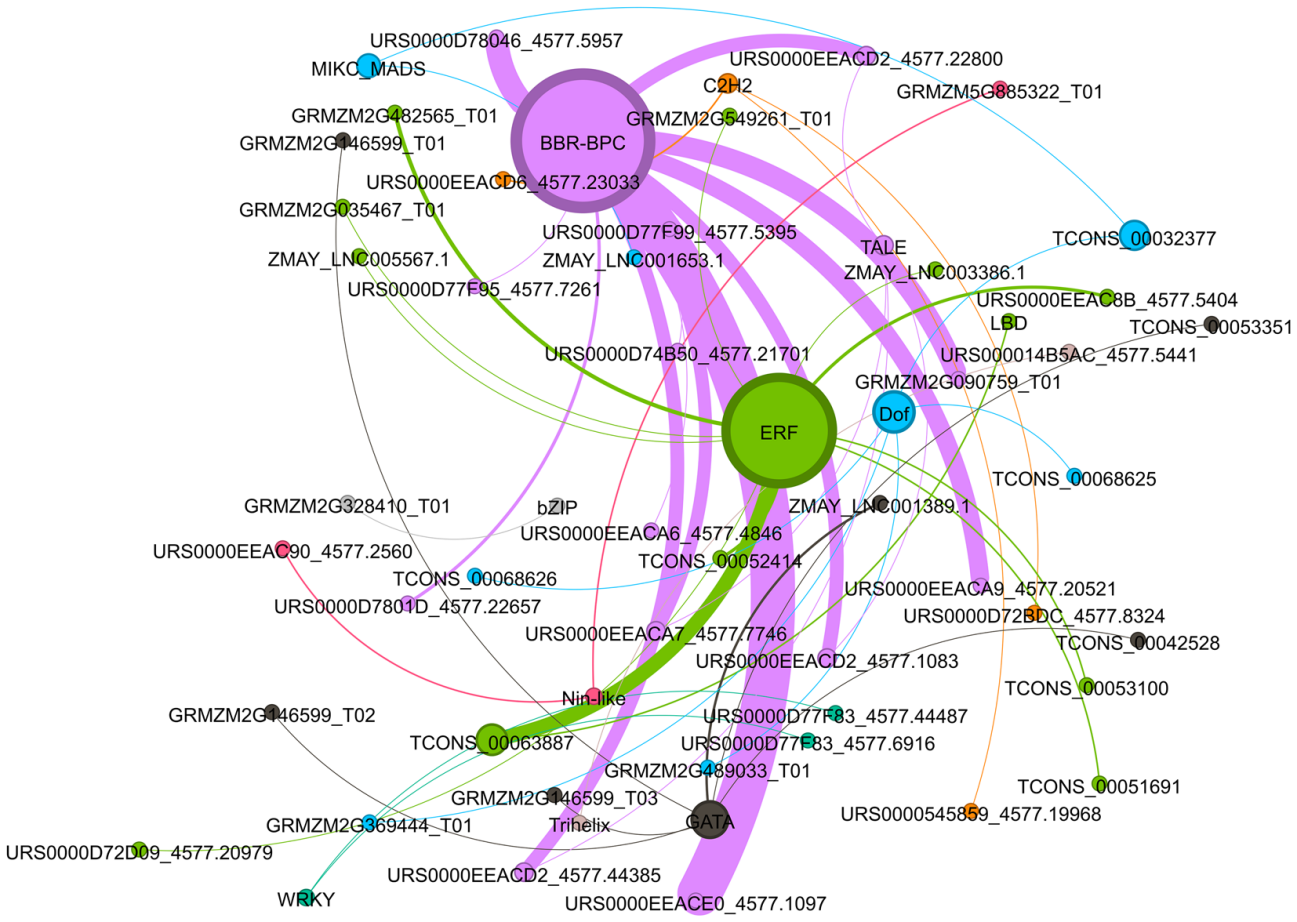
Interaction network showing association of lncRNAs with transcription factors in *Z. mays*. The interaction network for *A. thaliana* and *O. sativa* are depicted in Fig. S9. Nodes represent transcription factors, and edges represent lncRNAs. Node size is proportional to the number of interactions. A thick edge indicates interaction that occurs together more often than those with a thinner edge. Different color lines are used to make the image visibly distinct.

The potential lncRNA targeted by miRNA for cleavage might enrich the transcriptional regulatory proteins to facilitate transcription at specific loci^28^. Hence, to examine the cellular transcriptional regulation by lncRNAs, an association of TFs in miRNA-targeted lncRNA was investigated. Out of 663, 266, and 557 lncRNAs, 145, 86, and 45 lncRNAs were found to be highly associated with different TFs in *A. thaliana*, *O. sativa*, and *Z. mays*, respectively (Supplementary File 4). In *A. thaliana*, 145 lncRNAs interacted with 16 families of TFs, with the highest percentage of association observed for ERF, GATA, and BBR-BPC (Fig. S9A, Supplementary File 4). Similarly, in *O. sativa*, 86 lncRNAs interacted with 11 families of TFs, with the maximum binding observed for ERF and BBR-BPC (Fig. S9B, Supplementary File 4). In the case of *Z. mays,* 45 lncRNAs were found to bind with 12 families of TFs, with BBR-BPC followed by ERF being the most associated family (Fig. 6, Supplementary File 4). Interestingly, among all three plant species, the highest association of lncRNAs was found with BBR-BPC and ERF, implying their potential association for governing the biological processes regulated by these TFs. All the lncRNAs which showed a binding affinity for TFs, their binding site position, motif sequences, and statistically significant values (q-value) are provided in supplementary file 4.

## Discussion

LncRNA has gained widespread attention in the animal and plant kingdoms due to its involvement in various molecular and biological processes and response to environmental stimuli. This makes it imperative to understand the characteristics and potential roles associated with plant lncRNAs. We analyzed lncRNA from *A. thaliana*, *O. sativa*, and *Z. mays* available in the PLncDB V2.0 database for a broader picture. LncRNA distribution revealed that they are dispersed throughout the chromosome, including in PCG-deserted regions such as centromeres and telomeres (Fig. 1A; Fig. S1)^29^. The lncRNA distribution in centromeric and telomeric regions enriched with TEs possibly suggests their co-functioning. The genic structure revealed average exons per lncRNA were found to be 1.4, 1.7, and 1.6, in contrast to the 5.89, 4.2, and 9.2 exons per PCG in *A. thaliana*, *O. sativa*, and *Z. mays*, respectively^30–32^. We found the average size of lncRNAs to be 765, 2539, and 2438 nucleotides, smaller than the average gene length of 2080, 2853, and 4187 nucleotides in *A. thaliana*, *O. sativa*, and *Z. mays*, respectively^30–32^. The more exons number possibly explains the increase in lncRNA gene length, which correlates with genome size. Along the line, a study in several plant species showed intron size positively associated with genome size, so the average gene length^33^. We hypothesize an increase in exon number so the intron leads to the complexity of lncRNA genes, which could be a potential factor for structural and functional variations and an impetus factor in the evolution of structural complexity in lncRNA genes. This hypothesis is supported by a study in animals from different lineages, which showed that the number of exons per gene, including intron and 3'UTR region, progressively expanded from invertebrate ancestors to vertebrates during evolution^34^.

Localization analysis revealed that lncRNAs are more abundant in the nucleus of *A. thaliana* and *O. sativa*, which is analogous to their abundance reported in *Drosophila* and humans^35,36^. However, lncRNA localization showed variability in different subcellular fractions, which could be due to the limitation of the tool used for the study (Fig. 2A). Since it is an emerging field, more precise tools for predicting localization are expected to be developed for predicting localization as new training datasets become available. It is worth mentioning that several studies in the animal kingdom suggest that cytoplasmic lncRNAs are more stable than their nuclear counterparts, which reflects the nature of their biological function^37^. The stability of nuclear lncRNAs echoes their role in regulating gene expression, transcriptional reprogramming through chromatin interactions, and remodeling in response to various external and internal stimuli, which are very dynamic^37–39^. Meanwhile, in the cytoplasm, they are predominantly involved in signal transduction pathways, post-transcriptional and post-translational modifications^40,41^.

The analysed lncRNAs showed enrichment in epigenetic states previously identified as hotspots for other ncRNAs^25^. LncRNAs regulate transcription through various mechanisms, one of which is by maintaining specific TFs at transcription regulatory elements. The prominent binding of TFs in the epigenetic states enriched for lncRNA transcribing regions indicates their potential to regulate TF activity and levels around gene regulatory elements. Several studies across the kingdom support this notion. For example, the lncRNA NORAD binds to PUMILIO proteins in response to DNA damage and regulates genomic stability in humans^42^. In embryonic stem cells, lncRNA contributes to the stable occupancy of the TF Yin-Yang 1 at gene regulatory elements^43^. In the fission yeast *S. pombe*, lncRNAs known as mlonRNAs (metabolic stress-induced lncRNAs) regulate organism response to stress by facilitating chromatin remodeling along the promoter of the fbp1 + gene and promote the association of the transcription factor Atf1 with its regulatory elements^44^.

Interestingly, lncRNAs of *A. thaliana* showed enrichment in ESs 21, 29, and 30 (Fig. 3A), and all these epigenetic states showed enriched binding for WRKY TFs. The WRKY TF family is known to modulate several plant processes and forms an integral component of signaling webs in plants. A single WRKY TF could regulate diverse responses and contribute to the repression and de-repression of vital plant processes^45^. It is essential to mention that epigenetic states of DNA are dynamic in a spatial–temporal manner. Therefore, comparing the lncRNAs identified in different tissues and conditions is not ideal for such analysis. Nevertheless, our study reporting the insights gained about the general correlation between epigenetic states and lncRNA transcribing regions is relevant and worth reporting. This analysis also serves as a basis for future studies investigating cell-tissue-specific epigenome associations with lncRNA transcribing.

TEs are dispersed across the genome with several known hotspots and significantly impact genome architecture and evolution, and their presence and activity can shape the diversity and complexity of genomes^46,47^. Our analysis showed the enrichment of lncRNAs in epigenetic states that overlap with TEs (Fig. 3); however, lncRNA do not encode TEs^48^. An attempt has been made in plants to establish the systematic relationship between ncRNAs and TEs; however, only a fraction of these were represented by lncRNAs^49^. The reported study used a limited dataset compared to our investigation, which analyses more extensively and systematically identified lncRNA and TE datasets. The representation of TE in the genome varies among plant species, with approximately 24%, 40%, and 90% of the genome consisting of TEs in *A. thaliana*, *O. sativa*, and *Z. mays*, respectively^50–52^. This indicates that the lncRNA-TE association has a linear correlation (Fig. 4B).

Franco-Zorrilla and colleagues first defined the target mimic in 2007 in gene regulation. They showed that Induced by Phosphate Starvation1 (IPS1), a lncRNA binds to ath-miR399 with interrupted pairing at the cleavage site of ath-miR399 in *A. thaliana*^20^. We identified 11, 12, and 9 endogenous target mimics, which act as a sponge for the miRNAs. In a comparable study in *Brassica rapa*, 15 lncRNAs possess endogenous target mimics were identified from 12,052 lncRNAs during different pollen developmental stages, out of which two were experimentally confirmed as target mimics for miR160^53^. Another study in tomatoes showed multiple lncRNAs act as endogenous target mimics for microRNAs and their association with the yellow leaf curl virus infection^54^. Our investigation revealed that the miRNAs and endogenous target mimic sites showed conserved features among the studied plants (Fig. 5A, Fig. S5). A study in *Z. mays* reported the conserved endogenous target mimic site (zma_eTM_miR528b-5p-19) in several lncRNAs, supporting our findings^55^.

Our analyses showed 54.4%, 40.8%, and 84% of known miRNAs target and cleave lncRNAs in *A. thaliana*, *O. sativa*, and *Z. mays*, respectively (Supplementary File 1). The high percentage of association between miRNAs and lncRNAs indicates a potential mutual regulation of biological processes. GO enrichment analysis of lncRNA-miRNA targeted genes showed enriched processes associated with reproductive organ development, response to light, and signal transductions in *A. thaliana*. Several studies on developmental and tissue-specific lncRNA identification in plant reproductive organs suggest their reproductive development-related functions (Fig. 5B); however, we still do not know well-characterized lncRNAs that play crucial roles in organogenesis in plants^53,56–58^. Conversely, in mammals, several well-characterized lncRNAs reported to play essential roles in organogenesis; for example, the lncRNA *Bvht* and *Fendrr* in cardiac development, *Linc-MD1* in myogenesis, *lincRNA-EPS* in erythroid differentiation and *TINCR* in keratinocyte differentiation^59^. Further, several genome-wide plant studies support our finding and highlight the lncRNA role in light and signal transduction^24,60,61^.

In *O. sativa*, our analysis suggests lncRNA involvement in flower development, transcription regulation, regulation of macromolecule biosynthetic processes, etc. (Fig. S6A). LncRNA role in flowering is reported widely in different plant species, including *A. thaliana*^62,63^, *O. sativa*^14,56,64^, *Cicer arietinum*^65^, *Solanum lycopersicum*^66^, and *Z. mays*^11^. Several well-known lncRNAs have been reported to regulate flowering in plants, including COLDAIR^15^, COOLAIR^62^, COLDWRAP^67^, and ASL^68^, which negatively regulate the *FLOWERING LOCUS C* (FLC), a master regulator of flowering initiation, whereas *MAF4* Antisense (MAS) positively regulates the FLC expression^69^. The circadian-regulated long non-coding RNA (*FLORE*), a natural antisense of *CDF5*, is known to repress several *CYCLING DOF FACTOR* genes and negatively regulate *FLOWERING LOCUS T* (*FT*) and consequently activate the *FT* to promote photoperiodic flowering^70^.

Enrichment analysis of lncRNA-miRNA targeted genes in *Z. mays* showed processes such as DNA repair, lignin metabolic and catabolic processes, and DNA biosynthesis enrichment (Fig. S6B). In plants, the role of lncRNA in DNA repair is not well known; however, in mammals, several well-characterized lncRNAs are reported to play a crucial role in DNA repair. For example, in humans, a lncRNA named DNA damage-sensitive RNA1 (DDSR1) plays a critical role in modulating DNA repair by homologous recombination^71^. The lncRNA, *HOTAIRM1* serves as an assembly scaffold for non-homologous end joining (NHEJ) factors (Upf1/SMG6) to DNA double-strand breaks and subsequently helps in DSB repair^72^. Another lncRNA *LINP1*, was reported to direct the NHEJ-mediated DNA repair by interacting with the NHEJ factor Ku70/Ku80 (Ku) and Ku complexes^73^. An extensive study in Moso bamboo (*Phyllostachys edulis*) from different tissues and treatments highlighted the lncRNAs role in the secondary cell wall biosynthesis pathway^74^.

The conserved molecular function of lncRNA in all three studied plants showed enrichment for sequence-specific DNA binding, DNA-binding transcription factor activity and transcription regulator activity which conjointly advocate their involvement in governing the gene regulation supported by several well-studied lncRNAs (Fig. S7)^75^. The molecular function of identified lncRNA-miRNA-mRNA highlights their role in kinase and transferase activity (Fig. S7; Supplementary File 3), which are reported for the functional aspect of lncRNA in different animal models^7^. The enrichment of transcriptional cis-regulatory region binding in lncRNAs has been reported in several organisms and demonstrated to modulate the expression of target genes^76^. Overall, the lncRNA-miRNA-mRNA interaction network revealed that lncRNAs are involved in several molecular and biological processes, including growth, developmental, signaling networks, genome regulation, and metabolic pathways, which different studies across the kingdom have supported^4,24^.

LncRNAs can bind to transcriptional regulatory proteins, which can regulate gene expression. Therefore, targeting lncRNAs that can bind to transcription factors through miRNA cleavage can directly influence the transcriptional landscape of organisms. Our analyses revealed BBR-BPC and ERF association with lncRNA in all three studied plants suggesting their potential and conserved role in governing the biological processes in plants. BASIC PENTACYSTEINE (BPC), also called BARLEY B-RECOMBINANT (BBR) family, encodes GAGA-motif binding factors that govern several biological processes in plants^77,78^. BPC generally recruits repressive proteins like polycomb repressive complexes (PRC) to GAGA motifs for the transcriptional repression of downstream target genes^79,80^. It has been shown that BPCs directly recruit PRC2 and catalyze the trimethylation of histone H3 at Lys27 at target genes^81^. A systematic analysis of multiple BPC genes revealed their pleiotropic effects on vegetative and reproductive development. Thus, the BPC TF family is an integral part of several biological processes essential for plant growth and development, which complements our enrichment analyses^77,78^.

The ERF family, a prominent plant-specific transcription factor family, governs multiple developmental and physiological processes^82^. A study on spinach has identified two lncRNAs, namely MSTRG.16566.1 and MSTRG.16121.1, as potential endogenous target mimics for miR172, which target three genes encoding AP2/ERF^83^. This study indirectly links the ERF with lncRNA. A total of 122 ERFs have been found in *A. thaliana*, while 139 and 166 ERFs homologs have been reported in *O. sativa* and *Z. mays*, respectively^82,84^. The ERF family of TF acts as an essential regulator in many biological and physiological processes, such as the establishment of the floral meristem, plant morphogenesis, responsive mechanisms to hormone stress signaling, coordination of stress signaling in response to wound repair mechanisms, signal transduction, and metabolite regulation^82–85^. Additionally, we found several other TFs, such as B3, bHLH, bZIP, NAC, C2H2, and WRKY, that interact with lncRNA and are known to regulate vegetative and reproductive growth, responses to the broad spectrum of stresses, phytohormonal regulation, defense signaling, etc.^86–89^.

## Conclusion

Despite lacking protein-coding capability, lncRNAs have become crucial players in plant gene regulation and cellular processes. Our analysis has revealed that the complexity of lncRNA transcripts increases with the complexity of genomes. The subcellular localization of lncRNAs predominantly in the cytoplasm and nucleus reflects their apparent site of action. The analysis of lncRNA-miRNA-mRNA networks and the functional annotation of Gene Ontology provide a deeper understanding of the biological processes associated with lncRNAs in different plants. We observed the conservation of several miRNAs targeted by endogenous target mimics of lncRNAs, indicating a conserved mechanism in plants for controlling gene expression by fine-tuning miRNA activity. Further, we found that lncRNAs exhibit a strong affinity for several transcription factors essential for plant growth and development across the three studied plant species. The binding sites of these known transcription factors in the lncRNAs provide valuable insights for deciphering their associated functions and narrowing down the approach required for functional characterization, thereby uncovering their potential roles.

LncRNAs overlapping with protein-coding genes poses a challenge when manipulating the lncRNAs in vivo without perturbing the genes on the opposite strand. However, having this information beforehand enables us to choose the appropriate approach for functional characterization. Understanding the functional significance of lncRNAs in plants holds immense potential for crop improvement, stress tolerance, and sustainable agriculture. Unraveling their intricate regulatory networks and deciphering their roles will offer valuable insights into plant biology and create new opportunities for manipulating plant traits to address the challenges of food security and environmental sustainability. This study contributes to the characterization of lncRNAs and establishes the groundwork for future investigations into their specific roles.

## Materials and methods

### Analysis of lncRNA characteristics features

The lncRNAs and their associated data for *A. thaliana*, *O. sativa*, and *Z. mays* were downloaded from PLncDB V2.0, a comprehensive plant lncRNA database^23^. We used TBtools interactive toolkit to visualize the distribution of lncRNAs across chromosomes^90^. The coordinates of lncRNA transcripts and chromosomes were used as inputs. Using the GTF and sequence files of lncRNAs, we determined the median transcript length, exon length, splice variants, and GC content of lncRNAs.

### Subcellular localization of lncRNAs

To comprehend the biological roles of lncRNAs, we determined their localization in different subcellular organelles. We used lncLocator, an ensemble classifier for determining the subcellular localizations of lncRNAs^91^. LncLocator utilizes k-mer and high-level abstraction features to construct a classifier that predicts subcellular localizations^91^. For the lncRNAs that showed localization in more than one cellular compartment, we considered the localization with the highest score for a subcellular compartment.

### Analysis of epigenetic features associated with lncRNAs

To analyze the epigenetic signatures and conserved features associated with lncRNAs, we used the epigenetic dataset of the Plant Chromatin State Database (PCSD)^25^. For the association between lncRNAs and epigenetic states, we mapped lncRNA genes onto different epigenetic states, which included 36, 38, and 26 chromatin states dispersed across the genome of *A. thaliana*, *O. sativa*, and *Z. mays*, respectively, using the PCSD web tool^25^. The lncRNA distribution in different epigenetic states were plotted using a pie chart.

### Association study between lncRNAs and transposable elements

To investigate the relationship between lncRNAs and TEs, we determined the overlap between the lncRNA transcribing regions and TEs using the bedtools intersect intervals^92^. For TEs, we used the Atlas of Plant Transposable Elements database (APTEdb), which has uniform annotation criteria for plant TE classification and categorizes them into LTR, LINE, SINE, TIR, MITE, Helitron, and other remaining categories^26^. We downloaded the TEs annotation data for *A. thaliana*, *O. sativa*, and *Z. mays* from APTEdb in GFF3 format for association analysis^26^.

### Analyses of endogenous target mimics

To determine the endogenous target mimics in the lncRNAs, we used the psMimic v1.1 tool with default parameters^27^. This tool identifies a motif in the target sequence complementary to the miRNA. However, this complementarity is disrupted by a bulge around the supposed cleavage site, a key feature for target mimic activity. We used mature miRNA and lncRNA fasta files as input files. The mature miRNA sequences of studied plant species were downloaded from miRBase (https://mirbase.org/).

### Phylogenetic analysis and conserved motifs

To study the phylogenetic analysis of miRNA and lncRNA sequences that possess the endogenous target mimic, we performed multiple sequence alignments separately for miRNA stem-loop and lncRNA sequences using T-Coffee^93^. The cladogram data were generated using the Neighbour-joining tree method without distance corrections and visualized using iTOL v6^94^. To identify conserved motifs, we used MEME with default parameters^95^. We uploaded the output .xml files generated by motif scans through the MEME suite to the iTOL tree for visualization.

### Analyses of lncRNA-miRNA-mRNA interactome networks

To identify miRNA targets in the lncRNAs, we used the psRNATarget. We identified miRNA–lncRNA interactome using the default parameters, except for a more stringent cutoff threshold for the expectation value (Expectation = 3)^96^. We also determined the potential target genes for miRNAs interacting with lncRNAs using the psRNATarget at default parameters and an expectation value of 3. The genes targeted by lncRNA-associated miRNA were used to perform GO enrichment analysis using ShinyGO v0.76^97^.

### Analyses of transcription factor binding sites in lncRNAs

We extracted the lncRNA sequences targeted and cleaved by miRNAs (determined by psRNATarget) and used them to identify the TF binding sites. To determine potential TFs binding to lncRNAs, we used the Binding Site Prediction tool of the Plant Transcriptional Regulatory Map^98^. We used PlantTFDB v5.0 to identify TF binding sites with a threshold P-value ≤ 1e−8. The interaction networks of TFs and lncRNAs were developed using Gephi 0.9.1^99^.

### Methodology workflow

We have summarize the primary toolsets employed for various analyses within the current study, aiming to provide an overarching framework that encapsulates our study. (Fig. S10).

### Supplementary Information

Supplementary Figures.

Supplementary Table S1.

Supplementary Information 3.

Supplementary Information 4.

Supplementary Information 5.

Supplementary Information 6.

## Data Availability

The datasets analysed during the current study are available at Zenodo (https://zenodo.org/) with ID 4,017,591 of PLncDB V2.0 database (https://doi.org/10.1093/nar/gkaa910).
