# Supplementary material for: Deciphering shared attributes of plant long non-coding RNAs through a comparative computational approach
Source: Sci Rep. 2023 Sep 12;13:15101. doi: 10.1038/s41598-023-42420-7 (PMC10497521; doi:10.1038/s41598-023-42420-7)
Supplement: Supplementary file 1 — Supplementary Figures. [file 41598_2023_42420_MOESM1_ESM.pdf]

Deciphering shared attributes of plant long non-coding RNAs through a comparative computational approach

Vikash Kumar Yadav[a#*], Siddhi Kashinath Jalmi[a], Shalini Tiwari[b], Savita Kerkar[a]

[#] Present Address: National Institute of Plant Genome Research, New Delhi, 110067, India

[a] School of Biological Sciences and Biotechnology, Goa University, Goa 403206, India

[b] Department of Biochemistry and Molecular Biology, Oklahoma State University, Stillwater, OK, USA

[*]**Corresponding author**

Vikash Kumar Yadav

National Institute of Plant Genome Research, New Delhi, 110067, India

Email: vikash.yadav@nipgr.ac.in

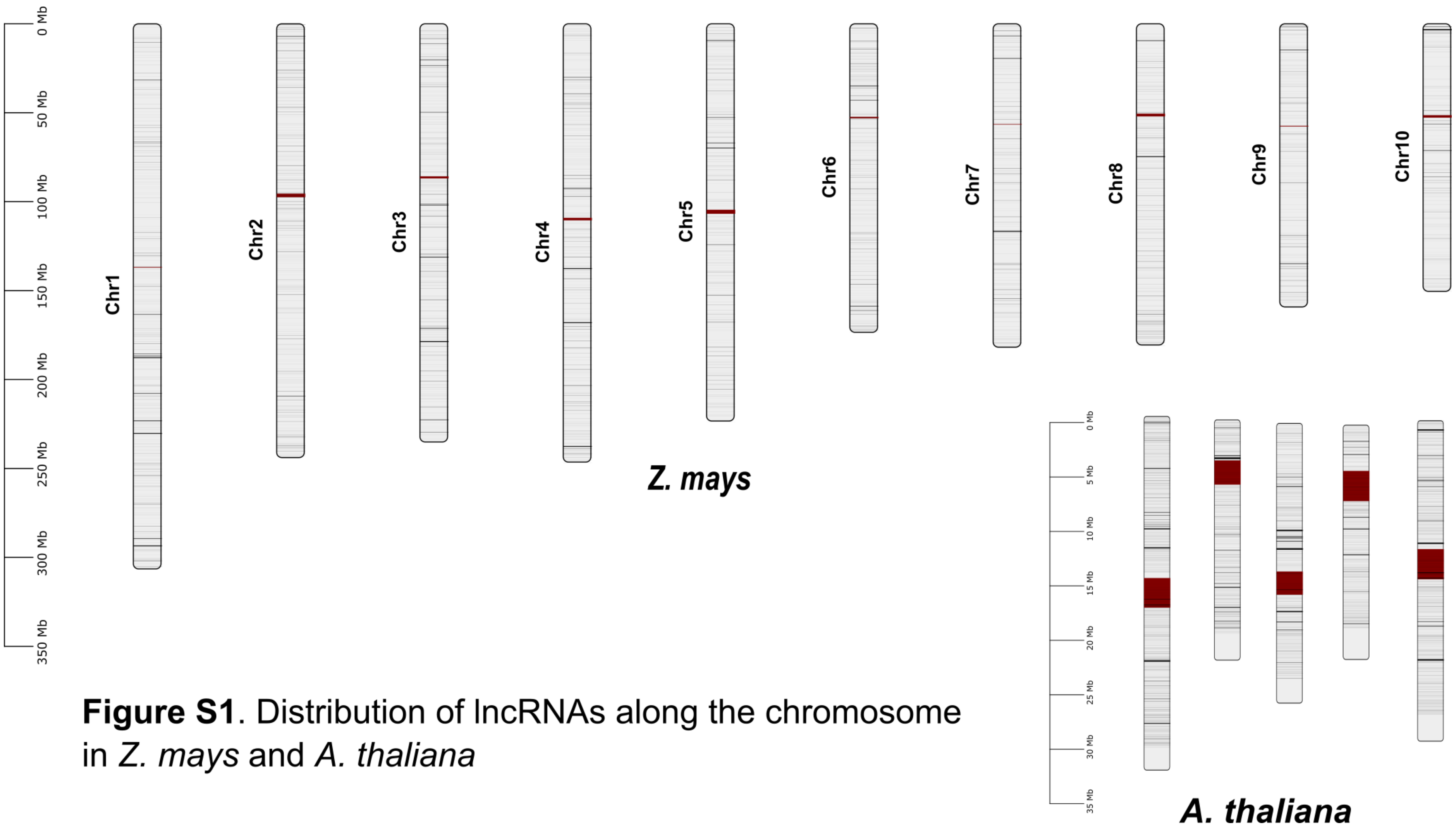

Figure S1. Distribution of lncRNAs along the chromosome in *Z. mays* and *A. thaliana*

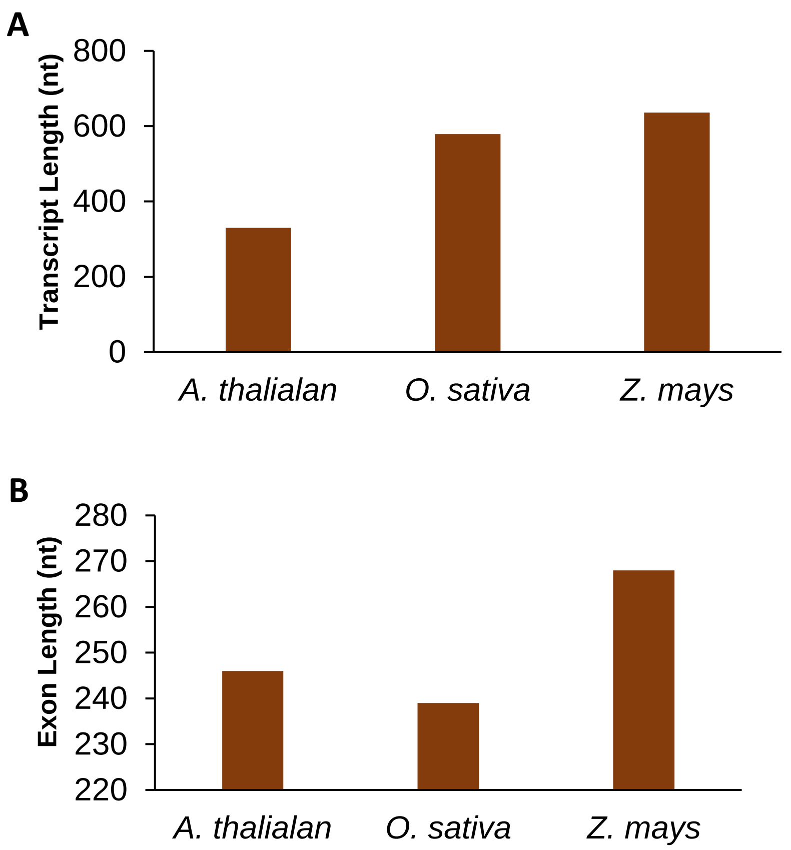

Figure S2. (A) The median size of full-length transcript of in *A. thaliana*, *O. sativa,* and *Z. mays*, respectively. **(B)** The median length of exon in *A. thaliana*, *O. sativa* and *Z. mays*, respectively.

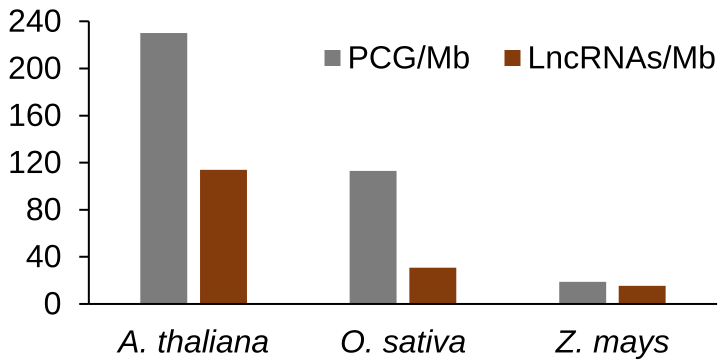

Figure S3. Bar graph showing the density of lncRNAs and protein coding genes per Mb of the genome.

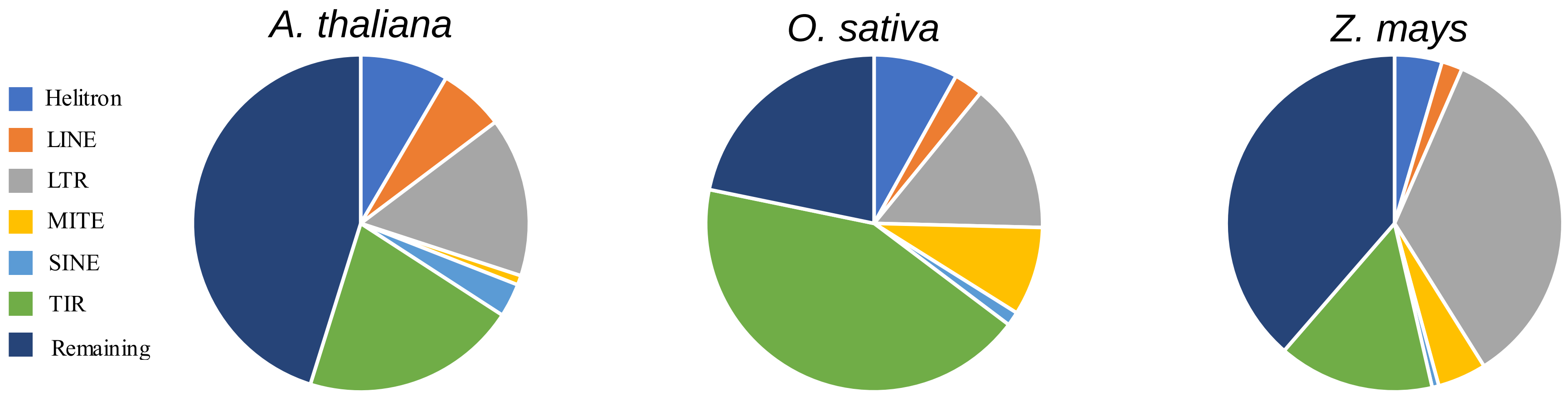

Transposable Elements	*A. thaliana*		*O. sativa*		*Z. mays*	
	**Genome-wide % TE distribution**	**% TE distribution in lncRNA**	**Genome-wide % TE distribution**	**% TE distribution in lncRNA**	**Genome-wide % TE distribution**	**% TE distribution in lncRNA**
SINE	3.21	2.17	1.36	1.42	0.66	0.45
LINE	6.30	5.93	2.79	2.59	2.00	1.58
LTR	15.32	15.56	14.51	14.59	**34.52**	**42.69**
MITE	0.91	0.92	8.52	7.55	4.67	2.77
Helitron	**8.43**	**13.98**	**8.10**	**9.30**	**4.57**	**9.59**
TIR	**20.68**	**16.56**	**42.95**	**40.43**	**14.95**	**9.77**
Remaining	45.16	44.88	21.77	24.39	38.64	33.15

Figure S4. (A) Distribution of different classes of TEs in the genome of *A. thaliana*, *O. sativa* and *Z. mays.* (B) Comparative table of TEs distribution in Genome and lncRNA transcribing regions

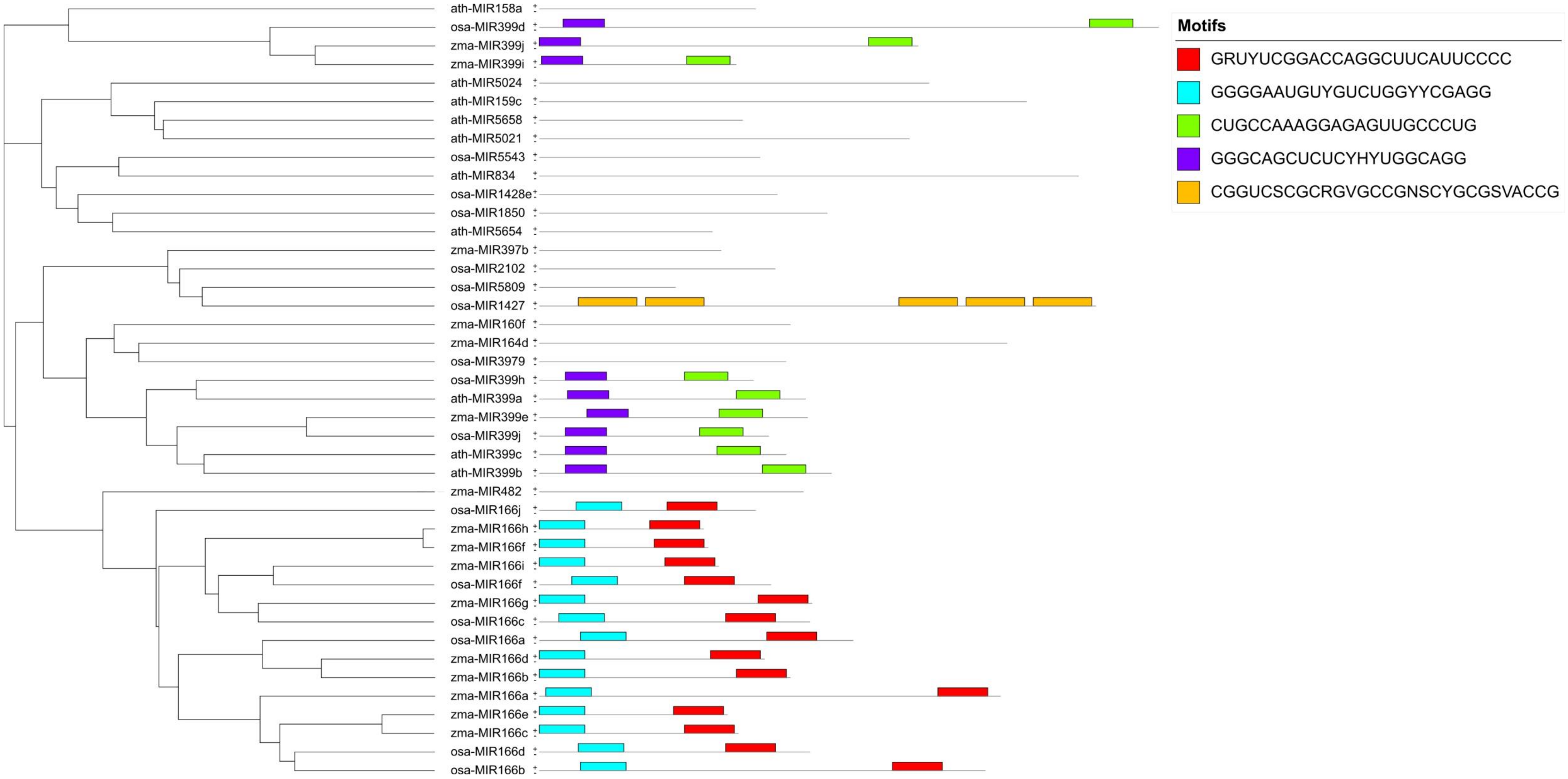

Figure S5: The cladogram showing the relatedness of miRNA targeted by lncRNA endogenous mimics among the *A. thaliana*, *O. sativa*, and *Z. mays.* The lncRNAs possessing the endogenous target mimic revealed conserved motifs in *A. thaliana*, *O. sativa*, and *Z. mays* lncRNAs, as determined by MEME.

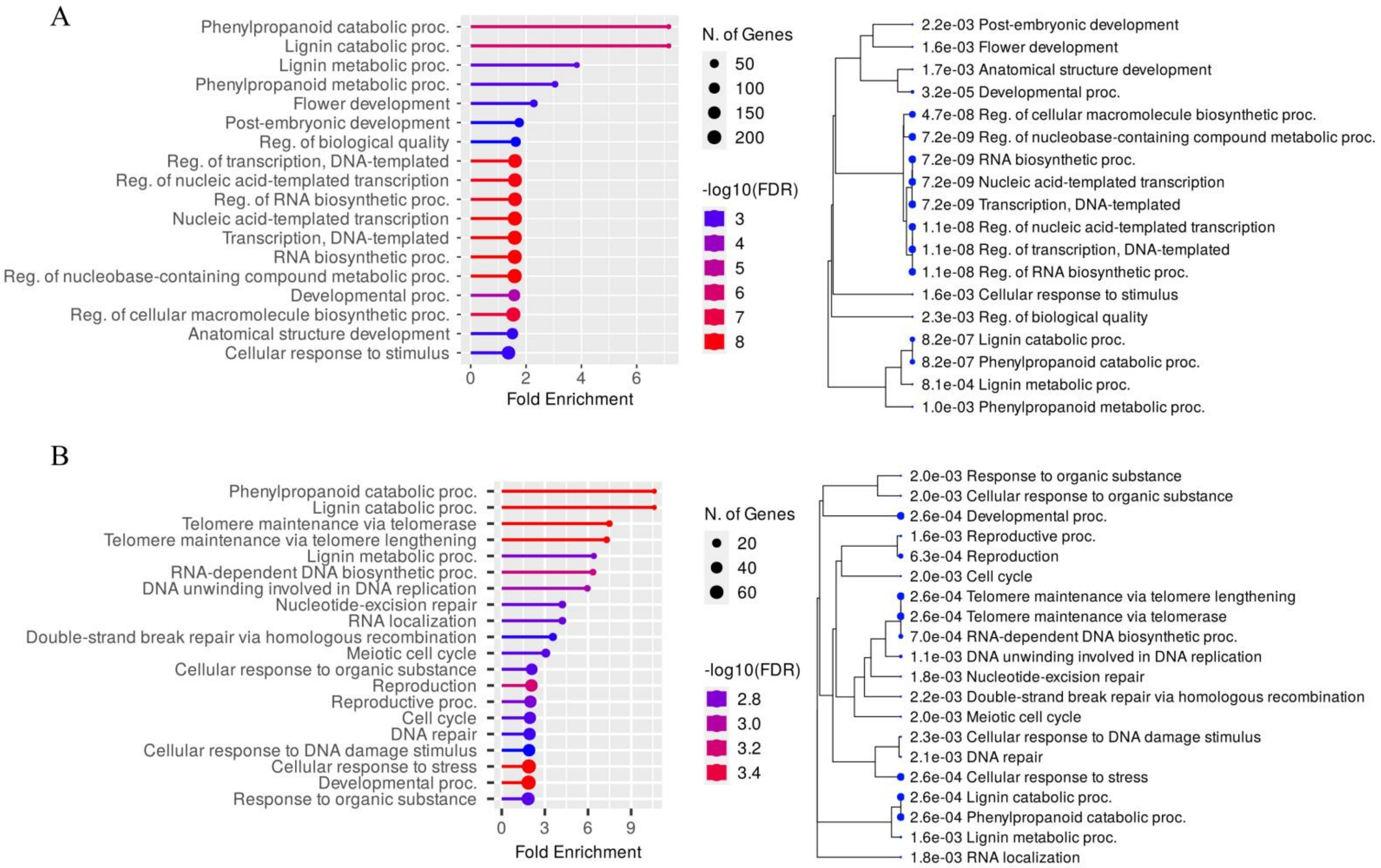

Figure S6: GO enrichment analysis showing the top 20 biological processes derived from lncRNA-associated miRNA-targeted genes in (A) *O. Sativa* and (B) *Z. mays*. The significant GO enrichment plots at $p < 0.05$ were depicted using ShinyGO v0.76. The fold enrichment of GO terms represents how drastically a particular pathway's genes are overrepresented.

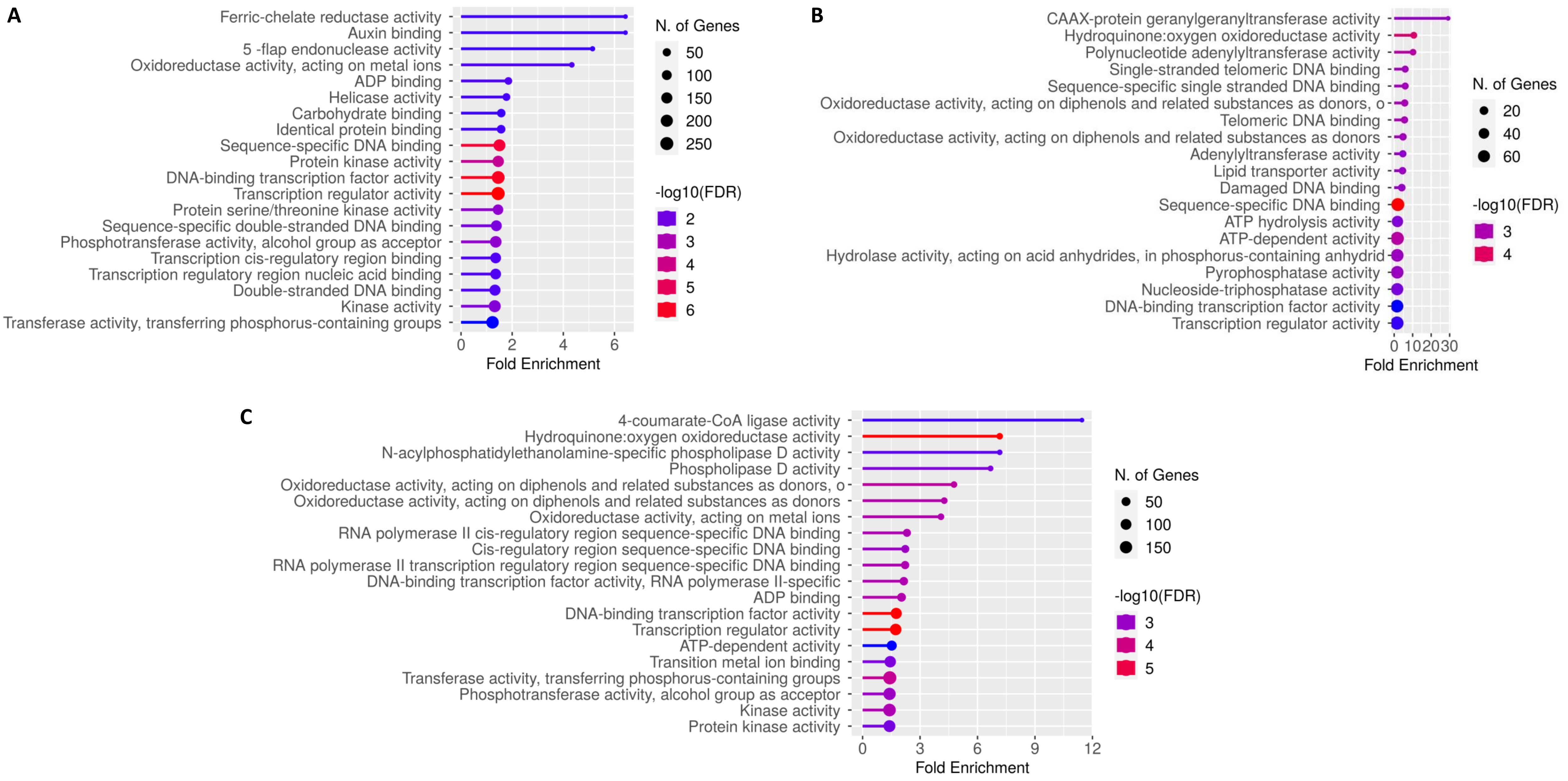

Figure S7: GO enrichment analysis showing the top 20 molecular function derived from lncRNA-associated miRNA-targeted genes in (A) *A. thaliana* (B) *Z. mays* and (C) *O. Sativa*.

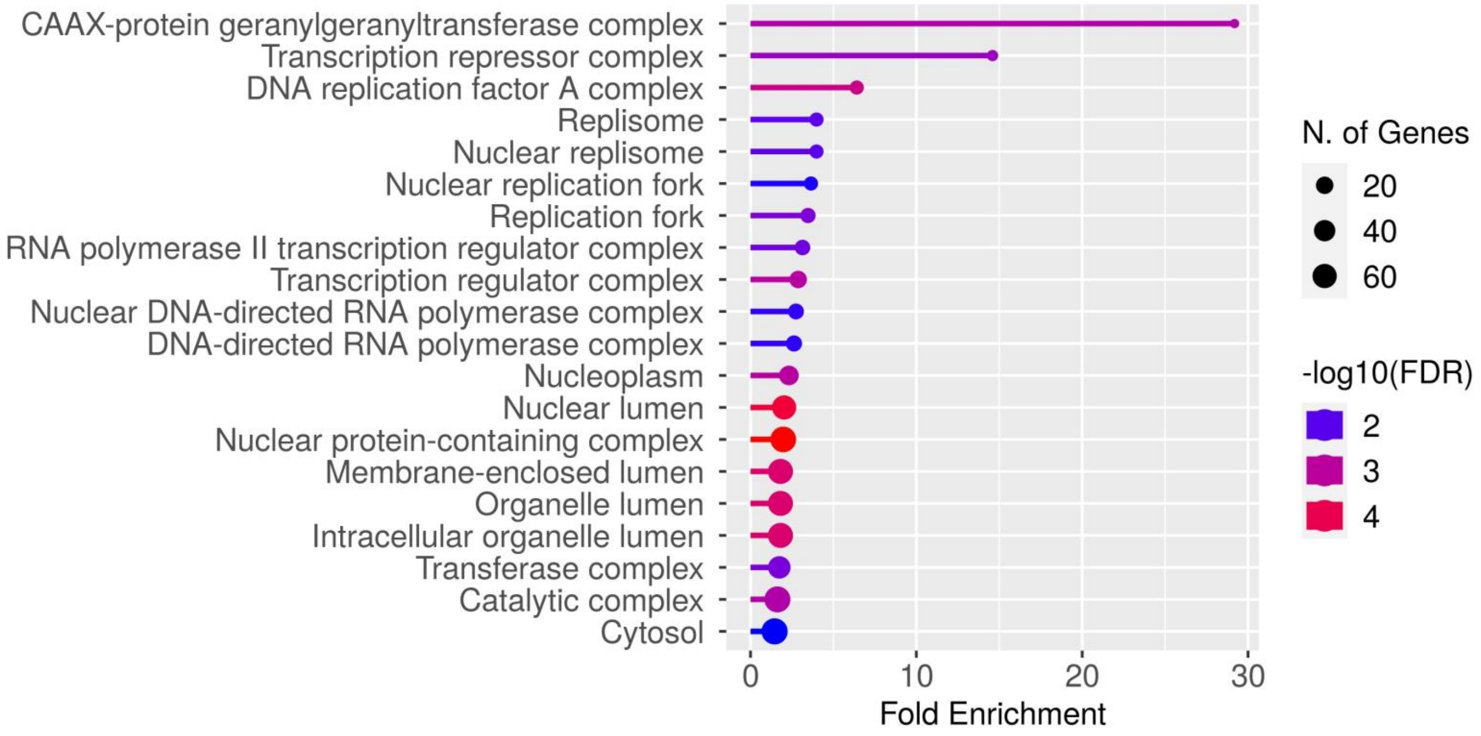

Figure S8: GO enrichment analysis showing the top 20 cellular component derived from lncRNA-associated miRNA-targeted genes in *Z. mays*

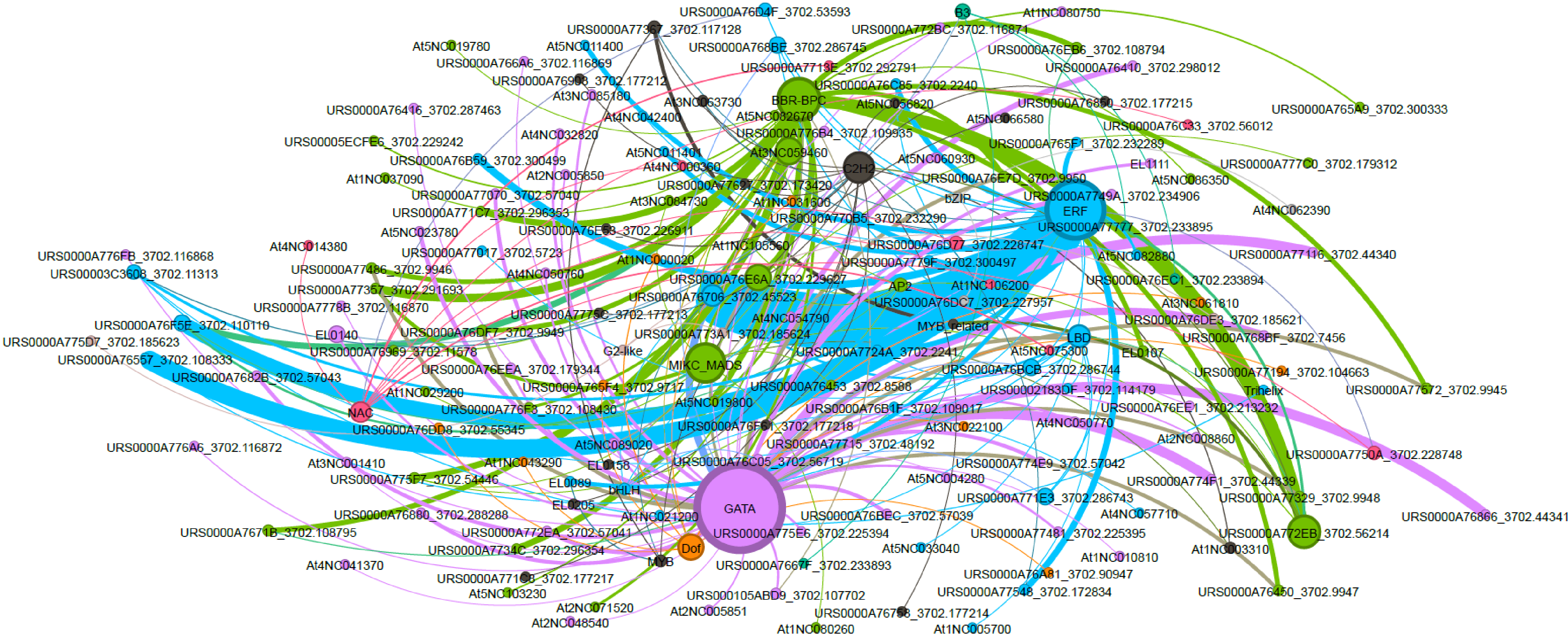

Figure S9A. Interaction network showing association of lncRNAs with transcription factors in *A. thaliana*. Nodes represent transcription factors and edges represents lncRNA. Node size is proportional to the number of interactions. Thick edge showed interaction occur together more often than those with a thinner edge.

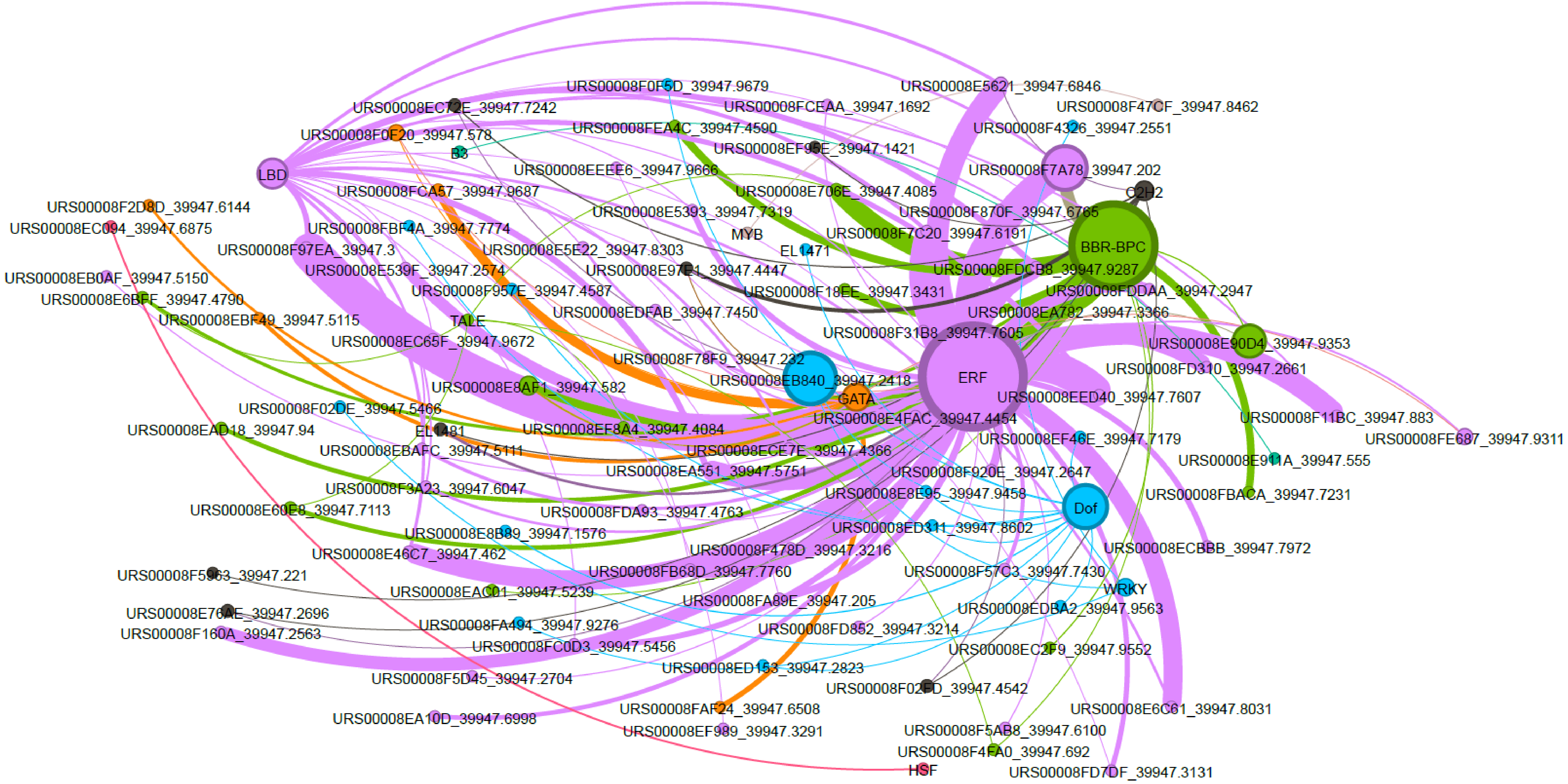

Figure S9B. Interaction network showing association of lncRNAs with transcription factors in *O. sativa*. Nodes represent transcription factors and edges represents lncRNA. Node size is proportional to the number of interactions. Thick edge showed interaction occur together more often than those with a thinner edge.

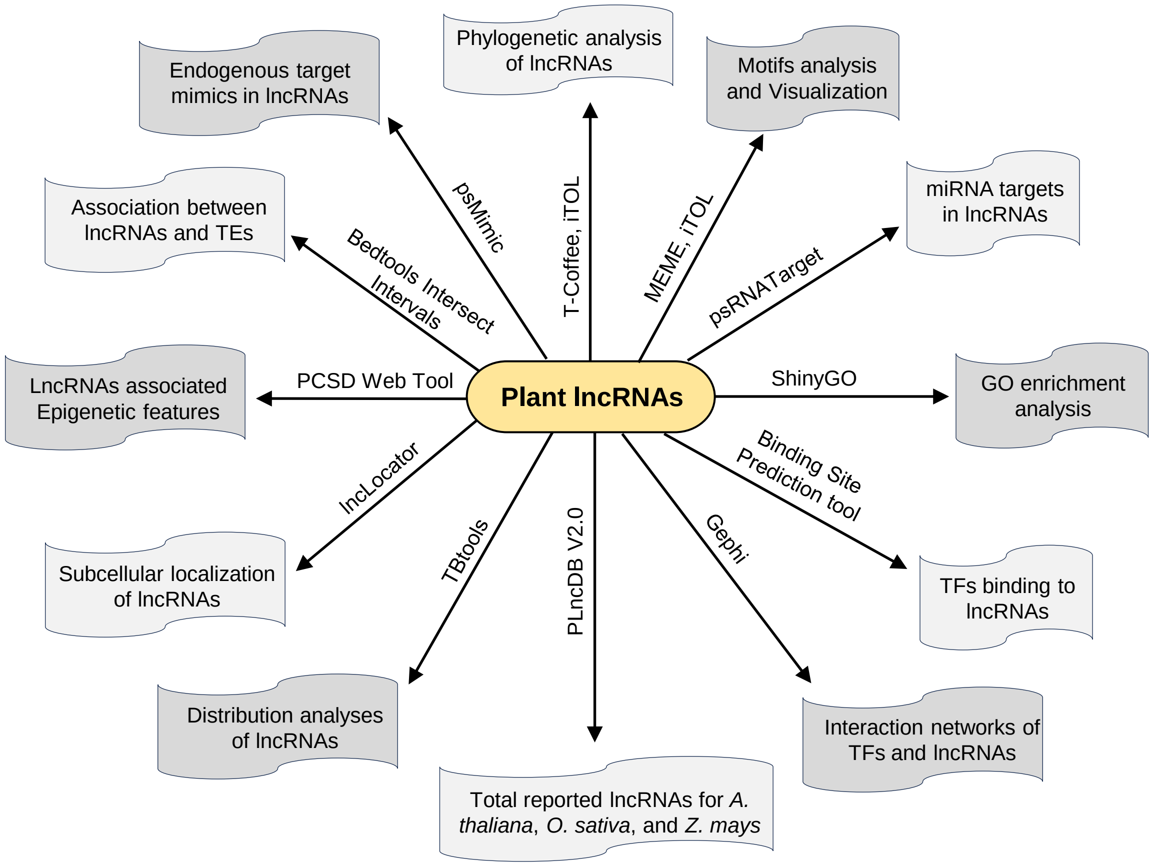

Figure S10. Schematic diagram showing an overview of computational tools used to study the characteristics and conserved features associated with plant lncRNAs.
