# Supplementary material for: Deciphering shared attributes of plant long non-coding RNAs through a comparative computational approach
Source: Sci Rep. 2023 Sep 12;13:15101. doi: 10.1038/s41598-023-42420-7 (PMC10497521; doi:10.1038/s41598-023-42420-7)
Supplement: Supplementary file 2 — Supplementary Table S1. [file 41598_2023_42420_MOESM2_ESM.pdf]

Table S1A: Prediction of the endogenous target mimics (eTM) in *A. thaliana* lncRNAs.

<table>
<tr><th>S.No</th><th>miRNA ID</th><th>lncRNA ID</th><th>eTM (Interrupted complimentary)</th></tr>
<tr><td>1</td><td>>ath-miR5021
MIMAT0020525</td><td>URS0000A76CDC_3702.226708</td><td>TGAGAAGAAG---AAGAAGAAAA
o||||||||| ||||||||||*
460 TCTCTTCTTCCGGTTCTTCTTTTG 437</td></tr>
<tr><td>2</td><td>>ath-miR158a-3p
MIMAT0000176</td><td>At2NC024550</td><td>TCCCAAATGT--A-GACAAAGCA
|||||||||| | :||||o||*
195 AGGGTTTACAATTATTGTTTAGTG 172</td></tr>
<tr><td>3</td><td>>ath-miR158a-3p
MIMAT0000176</td><td>URS0000A76F9E_3702.90588</td><td>TCCCAAATGT--A-GACAAAGCA
|||||||||| | :||||o||*
194 AGGGTTTACAATTATTGTTTAGTG 171</td></tr>
<tr><td>4</td><td>>ath-miR5658
MIMAT0022431</td><td>At3NC087030</td><td>ATGATGATGA---TGATGATGAAA
|||||||||| |o||||||||*
87 TACTACTACTAGAAATACTACTTTA 63</td></tr>
<tr><td>5</td><td>>ath-miR5654-3p
MIMAT0023005</td><td>URS0000A771E5_3702.56550</td><td>TGGAAGATGC--T-TTGGGATTTATT
|||||:|||| | ||||o||||||*
173 ACCTTTTACGAAACAACCGTAAATAAT 147</td></tr>
<tr><td>6</td><td>>ath-miR5024-3p
MIMAT0021048</td><td>URS000041CF79_3702.231333</td><td>CCGTATCTTG-G--CCTTGTCATT
|||||||||| | ||||||o|:*
947 GGCATAGAACTCTGGGAACAGAAGG 923</td></tr>
<tr><td>7</td><td>>ath-miR5024-3p
MIMAT0021048</td><td>URS00003FE7F7_3702.231334</td><td>CCGTATCTTG-G--CCTTGTCATT
|||||||||| | ||||||o|:*
1005 GGCATAGAACTCTGGGAACAGAAGG 981</td></tr>
<tr><td>8</td><td>>ath-miR834
MIMAT0004254</td><td>ATHA_LNC000008.1</td><td>TGGTAGCAGT---AGCGGTGGTAA
|||||||||| |||||||o||*
240 ACCATCGTCATAATCGCCACCCTTT 216</td></tr>
<tr><td>9</td><td>>ath-miR834
MIMAT0004254</td><td>ATHA_LNC000008.2</td><td>TGGTAGCAGT---AGCGGTGGTAA
|||||||||| |||||||o||*
152 ACCATCGTCATAATCGCCACCCTTT 128</td></tr>
<tr><td>10</td><td>>ath-miR399a
MIMAT0000951</td><td>At2NC062450</td><td>TGCCAAAGGA---GATTTGCCCTG
|||||||||| ||o|||||||:*
612 ACGGTTTCCTAGGCTCAACGGGATA 588</td></tr>
<tr><td>11</td><td>>ath-miR399b
MIMAT0000952</td><td>At2NC062450</td><td>TGCCAAAGGA---GAGTTGCCCTG
|||||||||| ||||||||||:*
612 ACGGTTTCCTAGGCTCAACGGGATA 588</td></tr>
<tr><td>12</td><td>>ath-miR159c
MIMAT0001015</td><td>At3NC063150</td><td>TTTGGATTG-AA--GGGAGCTCCT
||||:|||| || |::|||||||*
101 AAACTTAACATTACCTTTCGAGGAA 77</td></tr>
<tr><td>13</td><td>>ath-miR399c-3p
MIMAT0000953</td><td>At2NC062450</td><td>TGCCAAAGGA---GAGTTGCCCTG
|||||||||| ||||||||||:*
612 ACGGTTTCCTAGGCTCAACGGGATA 588</td></tr>
</table>

Table S1B: Prediction of the endogenous target mimics (eTM) in *O. sativa* lncRNAs.

S.No	miRNA ID	lncRNA ID	eTM (Interrupted complimentary)
1	>osa-miR5543<br>MIMAT0022179	OSAT_LNC000527.1<br>gene=OSAT_LNC000527	TATGAATGGT--A-TATTTTCTTG<br>|||||||||| | |o|||:||||*<br>832 ATACTTACCATATAAGAAAGGAACC 808
2	>osa-miR5543<br>MIMAT0022179	LOC_Os03r16696.1<br>gene=LOC_Os03r16696	TATGAATGGT--A-TATTTTCTTG<br>|||||||||| | |o|||:||||*<br>717 ATACTTACCATATAAGAAAGGAACC 693
3	>osa-miR3979-5p<br>MIMAT0019673	OSAT_LNC000630.1<br>gene=OSAT_LNC000630	TCTCTCTCTCC---CTTGAAGGC<br>||||||||||| ||:||||:o*<br>323 AGAGAGAGAGGTTAGAGCTTCTCA 300
4	>osa-miR399d<br>MIMAT0000987	EL1024	TGCCAAAGGA---GAGTTGCCCTG<br>|||||||||| :|||||||||:*<br>379 ACGGTTTCCTATGTTCAACGGGATG 355
5	>osa-miR399d<br>MIMAT0000987	URS00008F3C1B_39947.4237	TGCCAAAGGA---GAGTTGCCCTG<br>|||||||||| :|||||||||:*<br>301 ACGGTTTCCTATGTTCAACGGGATG 277
6	>osa-miR166b-3p<br>MIMAT0000636	URS00008EF6C7_39947.5681	TCGGACCAG---GCTTCATTCCCC<br>||||||||: ||||o|||||||*<br>292 AGCCTGGTTAAGCGAACTAAGGGGT 268
7	>osa-miR166a-3p<br>MIMAT0000635	URS00008EF6C7_39947.5681	TCGGACCAG---GCTTCATTCCCC<br>||||||||: ||||o|||||||*<br>292 AGCCTGGTTAAGCGAACTAAGGGGT 268
8	>osa-miR1850.2<br>MIMAT0009210	LOC_Os01r32775.1<br>gene=LOC_Os01r32775	TTGTGTGTGA---ACTAAACGTGG<br>|||||||||| |||o|||:|:|*<br>189 AACACACACTTGTTGACTTGTATCC 165
9	>osa-miR166d-3p<br>MIMAT0000638	URS00008EF6C7_39947.5681	TCGGACCAG---GCTTCATTCCCC<br>||||||||: ||||o|||||||*<br>292 AGCCTGGTTAAGCGAACTAAGGGGT 268
10	>osa-miR5809<br>MIMAT0023281	OSAT_LNC001283.1<br>gene=OSAT_LNC001283	TCGTCGCCGG---CGACCACAGC<br>:||||||||| |||||o||:|*<br>224 GGCAGCGGCCGTAGCTGGAGTTGG 201
11	>osa-miR399h<br>MIMAT0000991	URS00008EE938_39947.985	TGCCAAAGG--A-GACTTGCCCAG<br>||||||||| | ||||::||o||*<br>107 ACGGTTTCCGATCCTGAGTGGTTCC 83
12	>osa-miR1428e-3p<br>MIMAT0007785	LOC_Os04r56615.1<br>gene=LOC_Os04r56615	TAAGATAAT---GCCATGAATTTG<br>||||||||| ||||||o||||*<br>248 ATTCTATTAAATCGGTACTAAAACC 224
13	>osa-miR2102-5p<br>MIMAT0010068	URS00008E9D05_39947.4710	GGGCAAGCC---GCCGCCGCCAC<br>||||||:|| |||||||||o|*<br>629 CCCGTTTGGGATCGGCGGCGGCGG 606

<table>
<thead>
<tr><th>S.No</th><th>miRNA ID</th><th>lncRNA ID</th><th>eTM (Interrupted complimentary)</th></tr>
</thead>
<tbody>
<tr><td>14</td><td>>osa-miR1427
MIMAT0005961</td><td>OSAT_LNC000693.1
gene=OSAT_LNC000693</td><td>TGCGGAACCG--T-GCGGTGGCGC
|||||||||| | |||::||||o*
363 ACGCCTTGGCGAACCGCTGCCGCAG 339</td></tr>
<tr><td>15</td><td>>osa-miR166f
MIMAT0000640</td><td>URS00008EF6C7_39947.5681</td><td>TCGGACCAG---GCTTCATTCCCC
||||||||: ||||o|||||||*
292 AGCCTGGTTAAGCGAACTAAGGGGT 268</td></tr>
<tr><td>16</td><td>>osa-miR399j
MIMAT0000993</td><td>EL1024</td><td>TGCCAAAGGA---GAGTTGCCCTA
|||||||||| :||||||||||*
379 ACGGTTTCCTATGTTCAACGGGATG 355</td></tr>
<tr><td>17</td><td>>osa-miR399j
MIMAT0000993</td><td>URS00008F3C1B_39947.4237</td><td>TGCCAAAGGA---GAGTTGCCCTA
|||||||||| :||||||||||*
301 ACGGTTTCCTATGTTCAACGGGATG 277</td></tr>
<tr><td>18</td><td>>osa-miR166j-3p
MIMAT0001088</td><td>URS00008EF6C7_39947.5681</td><td>TCGGACCAG---GCTTCATTCCCC
||||||||: ||||o|||||||*
292 AGCCTGGTTAAGCGAACTAAGGGGT 268</td></tr>
<tr><td>19</td><td>>osa-miR166c-3p
MIMAT0000637</td><td>URS00008EF6C7_39947.5681</td><td>TCGGACCAG---GCTTCATTCCCC
||||||||: ||||o|||||||*
292 AGCCTGGTTAAGCGAACTAAGGGGT 268</td></tr>
</tbody>
</table>

Table S1C: Prediction of the endogenous target mimics (eTM) in *Z. mays* lncRNAs.

<table>
<tr><th>S.No</th><th>miRNA ID</th><th>lncRNA ID</th><th>eTM (Interrupted complimentary)</th></tr>
<tr><td>1</td><td>>zma-miR166a-3p
MIMAT0001375</td><td>URS000078222A_4577.3487</td><td>TCGGACCAGG---CTTCATTCCCC
||||||||:| |||o|||||||*
647 AGCCTGGTTCCGCGAACTAAGGGGG 623</td></tr>
<tr><td>2</td><td>>zma-miR166a-3p
MIMAT0001375</td><td>URS00007643DE_4577.3486</td><td>TCGGACCAGG---CTTCATTCCCC
||||||||:| |||o|||||||*
647 AGCCTGGTTCCGCGAACTAAGGGGG 623</td></tr>
<tr><td>3</td><td>>zma-miR166a-3p
MIMAT0001375</td><td>URS0000D769B0_4577.3488</td><td>TCGGACCAGG---CTTCATTCCCC
||||||||:| |||o|||||||*
47 AGCCTGGTTCCGCGAACTAAGGGGG 23</td></tr>
<tr><td>4</td><td>>zma-miR166a-3p
MIMAT0001375</td><td>ZMAY_LNC000547.1</td><td>TCGGACCAGG---CTTCATTCCCC
||||||||:| |||o|||||||*
633 AGCCTGGTTCTGCGAACTAAGGGGT 609</td></tr>
<tr><td>5</td><td>>zma-miR399i-3p
MIMAT0014024</td><td>URS0000D747F0_4577.109</td><td>TGCCAAAGGA---GAGTTGCCCTG
|||||||||| :|||||||||:*
426 ACGGTTTCCTATGTTCAACGGGATG 402</td></tr>
<tr><td>6</td><td>>zma-miR399e-3p
MIMAT0001708</td><td>URS0000D747F0_4577.109</td><td>TGCCAAAGGA---GAGTTGCCCTG
|||||||||| :|||||||||:*
426 ACGGTTTCCTATGTTCAACGGGATG 402</td></tr>
<tr><td>7</td><td>>zma-miR166b-3p
MIMAT0001382</td><td>URS000078222A_4577.3487</td><td>TCGGACCAGG---CTTCATTCCC
||||||||:| |||o||||||*
647 AGCCTGGTTCCGCGAACTAAGGGG 624</td></tr>
<tr><td>8</td><td>>zma-miR166b-3p
MIMAT0001382</td><td>URS00007643DE_4577.3486</td><td>TCGGACCAGG---CTTCATTCCC
||||||||:| |||o||||||*
647 AGCCTGGTTCCGCGAACTAAGGGG 624</td></tr>
<tr><td>9</td><td>>zma-miR166b-3p
MIMAT0001382</td><td>URS0000D769B0_4577.3488</td><td>TCGGACCAGG---CTTCATTCCC
||||||||:| |||o||||||*
47 AGCCTGGTTCCGCGAACTAAGGGG 24</td></tr>
<tr><td>10</td><td>>zma-miR166b-3p
MIMAT0001382</td><td>ZMAY_LNC000547.1</td><td>TCGGACCAGG---CTTCATTCCC
||||||||:| |||o||||||*
633 AGCCTGGTTCTGCGAACTAAGGGG 610</td></tr>
<tr><td>11</td><td>>zma-miR166e
MIMAT0001378</td><td>URS000078222A_4577.3487</td><td>TCGGACCAGG---CTTCATTCCC
||||||||:| |||o||||||*
647 AGCCTGGTTCCGCGAACTAAGGGG 624</td></tr>
<tr><td>12</td><td>>zma-miR166e
MIMAT0001378</td><td>URS00007643DE_4577.3486</td><td>TCGGACCAGG---CTTCATTCCC
||||||||:| |||o||||||*
647 AGCCTGGTTCCGCGAACTAAGGGG 624</td></tr>
<tr><td>13</td><td>>zma-miR166e
MIMAT0001378</td><td>URS0000D769B0_4577.3488</td><td>TCGGACCAGG---CTTCATTCCC
||||||||:| |||o||||||*
47 AGCCTGGTTCCGCGAACTAAGGGG 24</td></tr>
</table>

<table>
<tr><th>S.No</th><th>miRNA ID</th><th>lncRNA ID</th><th>eTM (Interrupted complimentary)</th></tr>
<tr><td>14</td><td>>zma-miR166e
MIMAT0001378</td><td>ZMAY_LNC000547.1</td><td>TCGGACCAGG---CTTCATTCCC
||||||||:| |||o||||||*
633 AGCCTGGTTCTGCGAACTAAGGGG 610</td></tr>
<tr><td>15</td><td>>zma-miR160f-5p
MIMAT0001750</td><td>URS0000D76A9A_4577.21215</td><td>TGCCTGGCT--C-CCTGTATGCCG
||||||||| | |o|||||||||*
406 ACGGACCGAAAGGGTACATACGGCG 382</td></tr>
<tr><td>16</td><td>>zma-miR166h-3p
MIMAT0001377</td><td>URS000078222A_4577.3487</td><td>TCGGACCAGG---CTTCATTCCC
||||||||:| |||o||||||*
647 AGCCTGGTTCCGCGAACTAAGGGG 624</td></tr>
<tr><td>17</td><td>>zma-miR166h-3p
MIMAT0001377</td><td>URS00007643DE_4577.3486</td><td>TCGGACCAGG---CTTCATTCCC
||||||||:| |||o||||||*
647 AGCCTGGTTCCGCGAACTAAGGGG 624</td></tr>
<tr><td>18</td><td>>zma-miR166h-3p
MIMAT0001377</td><td>URS0000D769B0_4577.3488</td><td>TCGGACCAGG---CTTCATTCCC
||||||||:| |||o||||||*
47 AGCCTGGTTCCGCGAACTAAGGGG 24</td></tr>
<tr><td>19</td><td>>zma-miR166h-3p
MIMAT0001377</td><td>ZMAY_LNC000547.1</td><td>TCGGACCAGG---CTTCATTCCC
||||||||:| |||o||||||*
633 AGCCTGGTTCTGCGAACTAAGGGG 610</td></tr>
<tr><td>20</td><td>>zma-miR166d-3p
MIMAT0001384</td><td>URS000078222A_4577.3487</td><td>TCGGACCAGG---CTTCATTCCC
||||||||:| |||o||||||*
647 AGCCTGGTTCCGCGAACTAAGGGG 624</td></tr>
<tr><td>21</td><td>>zma-miR166d-3p
MIMAT0001384</td><td>URS00007643DE_4577.3486</td><td>TCGGACCAGG---CTTCATTCCC
||||||||:| |||o||||||*
647 AGCCTGGTTCCGCGAACTAAGGGG 624</td></tr>
<tr><td>22</td><td>>zma-miR166d-3p
MIMAT0001384</td><td>URS0000D769B0_4577.3488</td><td>TCGGACCAGG---CTTCATTCCC
||||||||:| |||o||||||*
47 AGCCTGGTTCCGCGAACTAAGGGG 24</td></tr>
<tr><td>23</td><td>>zma-miR166d-3p
MIMAT0001384</td><td>ZMAY_LNC000547.1</td><td>TCGGACCAGG---CTTCATTCCC
||||||||:| |||o||||||*
633 AGCCTGGTTCTGCGAACTAAGGGG 610</td></tr>
<tr><td>24</td><td>>zma-miR482-5p
MIMAT0015361</td><td>GRMZM2G163636_T01</td><td>TGGGAGATGAA---GGAGCCTT
o||||||:||| ||||||||*
570 TCCCTCTGCTTCCGCCTCGGAAG 548</td></tr>
<tr><td>25</td><td>>zma-miR397b-3p
MIMAT0015353</td><td>GRMZM5G857327_T01</td><td>CCAGCGCTG--C-ACTCAATTACG
|||o||||| | |||||||||||*
118 GGTGGCGACTAGGTGAGTTAATGCG 94</td></tr>
<tr><td>26</td><td>>zma-miR166f
MIMAT0001380</td><td>URS000078222A_4577.3487</td><td>TCGGACCAGG---CTTCATTCCC
||||||||:| |||o||||||*
647 AGCCTGGTTCCGCGAACTAAGGGG 624</td></tr>
</table>

<table>
<tr><th>S.No</th><th>miRNA ID</th><th>lncRNA ID</th><th>eTM (Interrupted complimentary)</th></tr>
<tr><td>27</td><td>>zma-miR166f
MIMAT0001380</td><td>URS00007643DE_4577.3486</td><td>TCGGACCAGG---CTTCATTCCC
||||||||:| |||o||||||*
647 AGCCTGGTTCCGCGAACTAAGGGG 624</td></tr>
<tr><td>28</td><td>>zma-miR166f
MIMAT0001380</td><td>URS0000D769B0_4577.3488</td><td>TCGGACCAGG---CTTCATTCCC
||||||||:| |||o||||||*
47 AGCCTGGTTCCGCGAACTAAGGGG 24</td></tr>
<tr><td>29</td><td>>zma-miR166f
MIMAT0001380</td><td>ZMAY_LNC000547.1</td><td>TCGGACCAGG---CTTCATTCCC
||||||||:| |||o||||||*
633 AGCCTGGTTCTGCGAACTAAGGGG 610</td></tr>
<tr><td>30</td><td>>zma-miR166g-3p
MIMAT0001381</td><td>URS000078222A_4577.3487</td><td>TCGGACCAGG---CTTCATTCCC
||||||||:| |||o||||||*
647 AGCCTGGTTCCGCGAACTAAGGGG 624</td></tr>
<tr><td>31</td><td>>zma-miR166g-3p
MIMAT0001381</td><td>URS00007643DE_4577.3486</td><td>TCGGACCAGG---CTTCATTCCC
||||||||:| |||o||||||*
647 AGCCTGGTTCCGCGAACTAAGGGG 624</td></tr>
<tr><td>32</td><td>>zma-miR166g-3p
MIMAT0001381</td><td>URS0000D769B0_4577.3488</td><td>TCGGACCAGG---CTTCATTCCC
||||||||:| |||o||||||*
47 AGCCTGGTTCCGCGAACTAAGGGG 24</td></tr>
<tr><td>33</td><td>>zma-miR166g-3p
MIMAT0001381</td><td>ZMAY_LNC000547.1</td><td>TCGGACCAGG---CTTCATTCCC
||||||||:| |||o||||||*
633 AGCCTGGTTCTGCGAACTAAGGGG 610</td></tr>
<tr><td>34</td><td>>zma-miR166i-3p
MIMAT0001379</td><td>URS000078222A_4577.3487</td><td>TCGGACCAGG---CTTCATTCCC
||||||||:| |||o||||||*
647 AGCCTGGTTCCGCGAACTAAGGGG 624</td></tr>
<tr><td>35</td><td>>zma-miR166i-3p
MIMAT0001379</td><td>URS00007643DE_4577.3486</td><td>TCGGACCAGG---CTTCATTCCC
||||||||:| |||o||||||*
647 AGCCTGGTTCCGCGAACTAAGGGG 624</td></tr>
<tr><td>36</td><td>>zma-miR166i-3p
MIMAT0001379</td><td>URS0000D769B0_4577.3488</td><td>TCGGACCAGG---CTTCATTCCC
||||||||:| |||o||||||*
47 AGCCTGGTTCCGCGAACTAAGGGG 24</td></tr>
<tr><td>37</td><td>>zma-miR166i-3p
MIMAT0001379</td><td>ZMAY_LNC000547.1</td><td>TCGGACCAGG---CTTCATTCCC
||||||||:| |||o||||||*
633 AGCCTGGTTCTGCGAACTAAGGGG 610</td></tr>
<tr><td>38</td><td>>zma-miR399j-3p
MIMAT0014025</td><td>URS0000D747F0_4577.109</td><td>TGCCAAAGGA---GAGTTGCCCTG
|||||||||| :|||||||||:*
426 ACGGTTTCCTATGTTCAACGGGATG 402</td></tr>
<tr><td>39</td><td>>zma-miR166c-3p
MIMAT0001383</td><td>URS000078222A_4577.3487</td><td>TCGGACCAGG---CTTCATTCCC
||||||||:| |||o||||||*
647 AGCCTGGTTCCGCGAACTAAGGGG 624</td></tr>
</table>

S.No	miRNA ID	lncRNA ID	eTM (Interrupted complimentary)
40	>zma-miR166c-3p<br>MIMAT0001383	URS00007643DE_4577.3486	TCGGACCAGG---CTTCATTCCC ||||||||:| |||o||||||*<br>647 AGCCTGGTTCCGCGAACTAAGGGG 624
41	>zma-miR166c-3p<br>MIMAT0001383	URS0000D769B0_4577.3488	TCGGACCAGG---CTTCATTCCC ||||||||:| |||o||||||*<br>47 AGCCTGGTTCCGCGAACTAAGGGG 24
42	>zma-miR166c-3p<br>MIMAT0001383	ZMAY_LNC000547.1	TCGGACCAGG---CTTCATTCCC ||||||||:| |||o||||||*<br>633 AGCCTGGTTCTGCGAACTAAGGGG 610
43	>zma-miR164d-3p<br>MIMAT0015137	TCONS_00088581	CACGTGGTCTC---CTTCTCCAT |||:||||||| |o||||||o<br>316 GTGTACCAGAGTTAGTAGAGGTT 294
